# An Iontronic Bimodal Afferent Nerve for Cross‐Modal Neuromorphic Fusion and Robotic Sorting

**DOI:** 10.1002/advs.78621

**Published:** 2026-11-10

**Authors:** Mengkang Deng, Wenxin Cao, Xinci Gong, Jiangdong Gong, Hang Xu, Wenjing Wang, Chentao Du, Fei Wang, Zhiguang Liu, Wentao Xu, Tingrui Pan

**Affiliations:** ^1^ School of Biomedical Engineering University of Science and Technology of China Hefei China; ^2^ Center For Intelligent Medical Equipment and Devices Suzhou Institute for Advanced Research University of Science and Technology of China Suzhou China; ^3^ Department of Applied Biology and Chemical Technology and Research Institute for Smart Energy The Hong Kong Polytechnic University Hong Kong China; ^4^ Department of Precision Machinery and Instrumentation University of Science and Technology of China Hefei China; ^5^ Department of Biomedical Engineering City University of Hong Kong Hong Kong China; ^6^ Institute of Photoelectronic Thin Film Devices and Technology Key Laboratory of Photoelectronic Thin Film Devices and Technology of Tianjin College of Electronic Information and Optical Engineering Nankai University Tianjin China; ^7^ Shenzhen International Graduate School Tsinghua University Shenzhen China

**Keywords:** artificial afferent nerve, crossmodal fusion, iontronic dual‐modal sensing, near‐sensor neuromorphic computing

## Abstract

Inspired by the multisensory perception and fusion strategy of the biological tactile system, we present a bimodal iontronic afferent nerve that integrates a flexible pressure–temperature sensor patch, pulse‐encoding electronics, an electrolyte‐gated synaptic transistor and a dual‐branch neuromorphic classification framework. The flexible sensor patch enables pressure and temperature perception over broad ranges, covering mechanical stimuli from 0 to 300 kPa and thermal stimuli from 0°C to 80°C. Rather than functioning as an isolated sensor, the system converts mechanical and thermal tactile cues into unified iontronic/spiking representations, enabling neuromorphic encoding, synaptic modulation and cross‐modal feature fusion. By integrating pressure and temperature features through a dual‐branch neuromorphic network, the system achieves 92.00% accuracy under within‐recording contiguous‐block validation with dual‐feature fusion and outperforming single‐modal classification. Finally, the integrated afferent nerve is applied to feed‐forward robotic tactile sorting, where objects with different color‐derived thermal states and mechanical stiffness are identified and classified. This work demonstrates a flexible bimodal neuromorphic tactile system capable of sensing, encoding, fusing and acting on multimodal tactile information, providing a promising route toward artificial skin for intelligent robotic perception.

## Graphical abstract

A flexible iontronic pressure–temperature sensor is integrated with pulse encoding and a multi‐gate SnO_2_ nanowire synaptic transistor to create a near‐sensor neuromorphic platform. Pressure and temperature cues are encoded, temporally integrated, and fused for accurate multimodal object recognition and robotic sorting, establishing a compact pathway from tactile sensing to neuromorphic perception and embodied intelligent action.

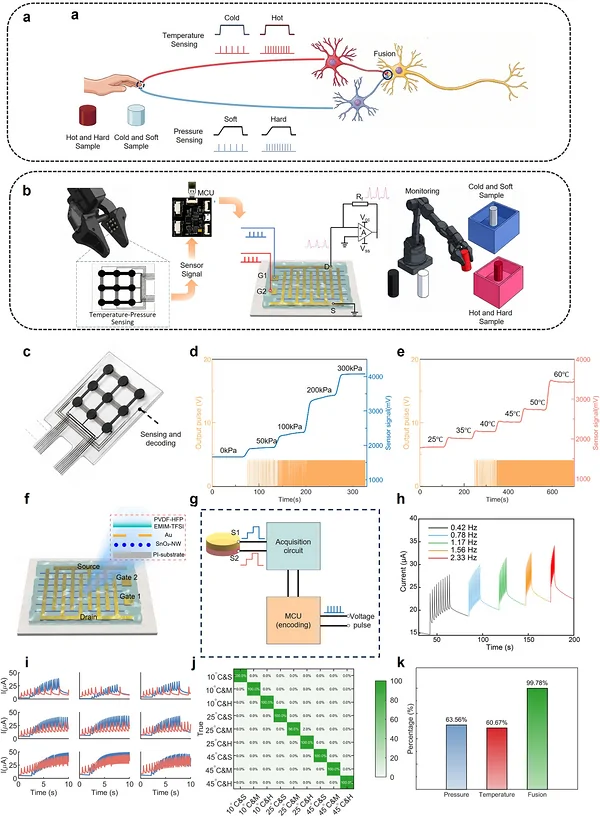

## 1 Introduction

The biological tactile system is one of the most sophisticated perception paradigms, where multimodal signals can be perceived spontaneously by various specific tactile receptors distributed within the skin [1]. These signals undergo neural fiber encoding, synaptic fusion, and hierarchical processing, ultimately forming accurate perceptions of object texture, hardness, or temperature, which support efficient environmental interaction and behavioral decision‐making of living organisms. Its key advantages lie in the parallel acquisition of multimodal signals, low‐power neural processing, and event‐driven real‐time response, providing a core bionic blueprint for the innovation of artificial perception electronics and systems [1, 2].

Facing the cutting‐edge development of embodied intelligence, fields such as robotics, electronic skin, human‐machine interaction, and intelligent prosthetics have an urgent demand for perception, computation and integration terminal systems [3]. Traditional perception architectures have long adopted a separated mode of sensor array, long‐distance signal transmission and central processor computing, which has inherent defects including data redundancy, transmission delay, high power consumption, bandwidth bottlenecks, and structural redundancy [4]. These limitations make it difficult to meet the essential application requirements of embodied intelligence for a low‐latency, highly autonomous, and self‐adaptive intelligent perception–decision pathway. Meanwhile, multimodal signals rely on backend algorithms for post‐fusion processing, and the ability of near‐sensor signal collaborative processing is lacking. This makes it difficult to achieve efficient collaboration between multimodal perception and computing, which greatly restricts the performance improvement of terminal intelligent perception systems [4, 5].

Neuromorphic perception technology, by simulating the multimodal perception and neural synaptic information processing mechanisms of the biological nervous system, deeply integrates sensing, encoding, storage, and computing functions, focusing on edge computing near the sensing end, and provides a key solution to address the pain points of traditional sensing and processing technologies [6, 7]. Among them, multimodal tactile perception fusion, as a core research direction of neuromorphic perception, emulates the cross‐modal information integration mechanism at the peripheral nerve level of biological touch, supports information processing close to the signal acquisition end, and implements an efficient intelligent perception paradigm of simultaneous detection and computing [5, 6].

Recent neuromorphic tactile systems have pursued several complementary routes toward the integration of sensing, neural encoding, memory, and robotic interaction. Flexible artificial sensory nerves have integrated tactile sensing with physical synaptic processing for material and temporal‐pattern recognition [5], while artificial organic afferent nerves have further enabled closed‐loop tactile feedback in robotic systems [8]. More tightly coupled device architectures, including mechano‐gated iontronic piezomemristors, have combined mechanical sensing and temporal‐tactile plasticity through a mechanogate, an oscillator module and a memristor [9]. In parallel, self‐powered neuromorphic touch systems based on triboelectric devices have been developed to reduce dependence on external power, although their integration, signal compatibility, and system‐level learning capabilities remain active challenges [10]. Recent studies have also extended neuromorphic processing toward multimodal perception and sensorimotor learning. Organic neuromorphic circuits have enabled robot learning through sensorimotor integration and maze navigation [11], as well as multimodal associative learning and behavioral conditioning through local adaptive processing [12]. Visual–tactile neuromorphic arrays and iontronic synapse‐fiber networks have demonstrated cross‐modal memory, spatiotemporal integration, and sensorimotor control [13, 14]. Meanwhile, physics‐based studies of organic artificial neurons have established important relationships among ionic transport, device parameters, excitability, and spiking behavior [15]. Printed iontronic robotic skins have also been combined with machine‐learning‐assisted recognition [16]. Collectively, these studies demonstrate major advances in self‐powered sensing, device‐level neuronal dynamics, adaptive learning, and closed‐loop feedback (Table S3). However, the integration of iontronic pressure–temperature sensing, modality‐specific spike encoding, electrolyte‐gated synaptic‐state generation, cross‐modal classification, and robotic sorting within one artificial afferent pathway remains underexplored.

In this study, we develop an iontronic‐transport artificial afferent nerve that integrates a flexible pressure‐temperature iontronic sensor, microcontroller‐based spike encoding, an electrolyte‐gated artificial synaptic device, and a dual‐feature neuromorphic classification framework. The sensor uses a polyelectrolyte elastomer (PEE)‐based pressure‐sensitive layer and a temperature‐sensitive layer in a compact multilayer architecture, enabling separately addressed mechanical and thermal readout [17, 18]. The pressure channel provides high linearity (R^2^ = 0.997) over 0–300 kPa, low drift (0.99%), rapid response (39.2 ms), and array‐level consistency (RSD = 4.8%), while the temperature channel provides stable thermal sensing (from 0 to 80°C) with limited pressure interference. Encoded pressure and temperature spikes are further processed by the SnO_2_ Nanowire (NW)‐based synaptic transistor to generate discriminative postsynaptic‐current features. By fusing pressure and temperature information, the system achieves high‐accuracy recognition and demonstrates robotic sorting of objects with coupled color‐derived thermal states and mechanical stiffness, establishing a hybrid hardware–software tactile pathway from iontronic reception to neuromorphic processing and robotic action.

## 2 Results and Discussion

### 2.1 Concept and Design of Iontronic‐Transport Afferent Nerve

Human somatosensation relies on distributed receptors in the skin to transduce external mechanical and thermal stimuli into electrical activity in afferent nerves [19]. During contact with objects, pressure, stiffness and temperature are not perceived as isolated physical quantities, but are encoded into temporally structured spike trains and transmitted through parallel neural pathways for downstream integration and decision‐making [20]. Such biological afferent systems provide an efficient strategy for early sensory coding, in which sensory transduction, spike generation and synaptic processing are spatially coupled [21] before high‐level perception is formed in the central nervous system.

Inspired by this sensory architecture, we designed an iontronic‐transport afferent nerve that integrates multimodal tactile sensing, spike encoding and synaptic signal processing in a hybrid hardware–software perception system (Figure 1). As illustrated in Figure 1a, biological pressure and temperature receptors convert external tactile inputs into neural firing patterns, where the firing frequency and temporal sequence carry information about stimulus modality and intensity [22]. To emulate this process, our artificial system uses an integrated iontronic pressure–temperature sensor [23] as the receptive terminal, a capacitance acquisition circuit and microcontroller unit as the encoding module, and an artificial synaptic transistor as the neuromorphic processing unit (Figure 1b) [24].

**FIGURE 1:**
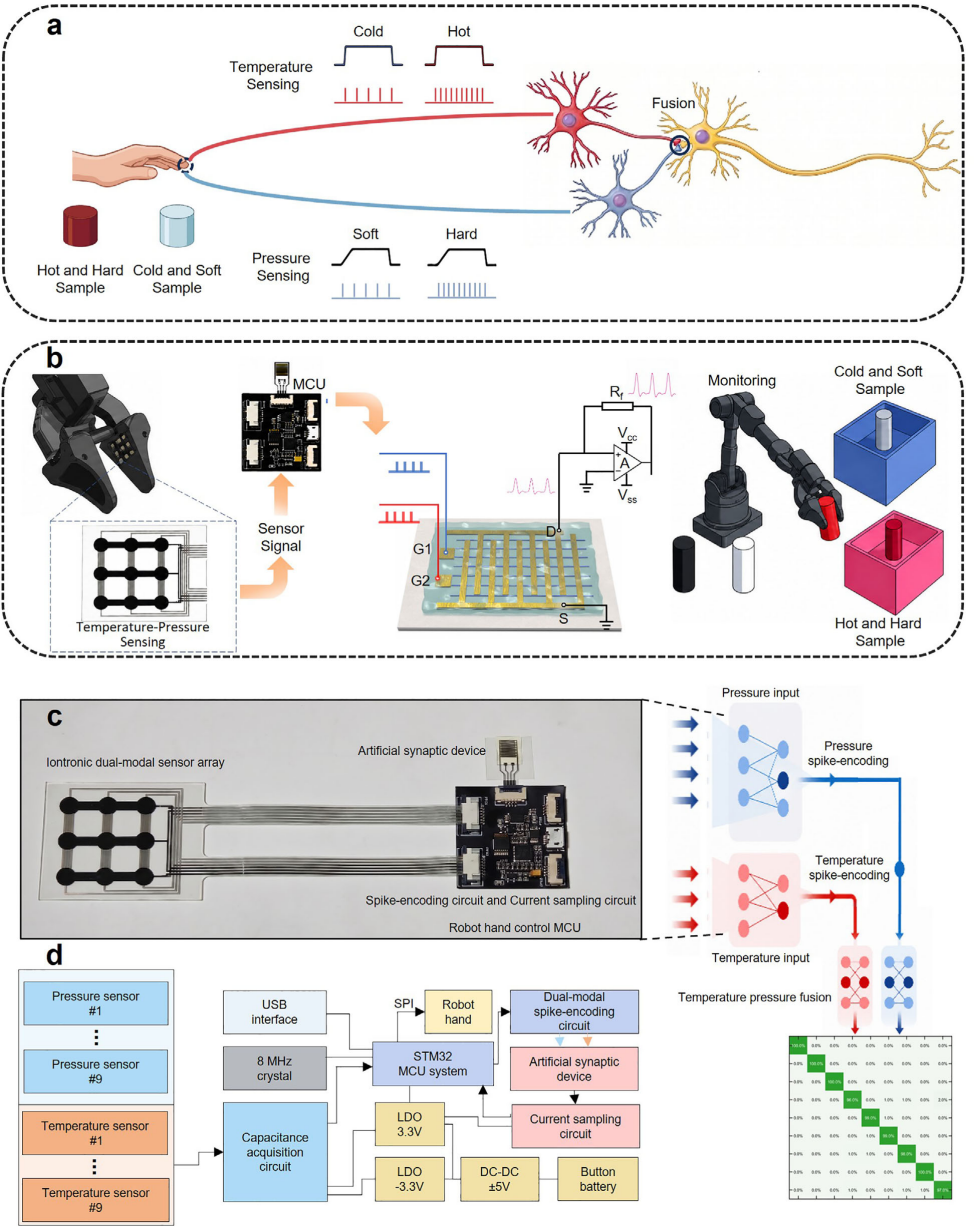
Concept and design of the iontronic‐transport afferent nerve for multimodal tactile perception. (a) Schematic illustration of biological somatosensory transduction and afferent transmission. External tactile stimuli with coupled mechanical and thermal attributes are detected by cutaneous sensory receptors and converted into neural spike trains. The encoded signals are transmitted through afferent nerve fibers to downstream neurons, where stimulus intensity and modality are represented by spike frequency and temporal firing patterns. (b) Bioinspired architecture of the artificial iontronic‐transport afferent nerve. The system consists of an integrated iontronic pressure–temperature sensor, a capacitance acquisition circuit, a microcontroller‐based spike‐encoding module, an artificial synaptic device and a current‐sampling circuit. Pressure‐ and temperature‐dependent sensor signals are converted into voltage pulse trains and then delivered to the artificial synapse for neuromorphic feature extraction. The processed output is further used to control a robotic arm for object recognition and sorting. (c) Schematic of the flexible integrated sensing platform, showing soft encapsulation, robust soft–flexible interconnects, soft sensor modules and wireless data transmission to a mobile interface. (d) Photograph and circuit‐level layout of the integrated hardware system, including the robot‐hand control MCU, spike‐encoding and current‐sampling circuit, artificial synaptic device, capacitance acquisition circuit and iontronic tactile sensor array. The block diagram summarizes the electrical modules used for sensor readout, pulse encoding, synaptic stimulation, current acquisition and robotic actuation.

In this artificial afferent pathway, pressure and temperature stimuli first modulate the electrical output of the iontronic sensor. The capacitance acquisition circuit converts the sensor response into voltage signals, which are subsequently transformed by the STM32‐based encoding module into discrete pulse trains. This encoding process mimics the transformation of receptor potentials into action‐potential‐like spike sequences in biological afferent nerves [25]. The encoded pressure and temperature pulses are then applied to the artificial synaptic device through dual‐gate terminals, where ion/electron coupling induces postsynaptic‐current responses with history‐dependent plasticity [26]. The physical synaptic device thereby performs a history‐dependent pulse‐to‐PSC transformation, and the resulting PSC traces are recorded by the current‐sampling circuit. Therefore, continuous analog tactile inputs are converted into neuromorphic spike streams and synaptic current states, enabling near‐sensor feature extraction before classification enabling MCU‐based spike encoding and physical synaptic‐state transformation near the sensing stage. Numerical PSC descriptor extraction, training‐set‐based normalization, dual‐branch feature fusion, classification, and class‐decision generation are subsequently performed offline in software rather than by the synaptic device or an on‐chip classifier [27].

The complete system was further implemented as an integrated flexible sensing and robotic‐interaction platform. The soft sensor module, encapsulation layer and soft–flexible interconnects provide mechanical compliance for tactile contact, while the acquisition, encoding and current‐sampling circuits allow conversion from external stimuli to synaptic outputs (Figure 1c, d). By linking the synaptic output with a robotic control module, the iontronic‐transport afferent nerve establishes a feed‐forward signal pathway from tactile reception to neuromorphic processing and robotic actuation. After classification, the predicted class label is transferred to the robot‐control MCU, which executes the corresponding sorting action. The system therefore establishes a feed‐forward perception‐to‐action pathway that combines physical sensing and synaptic processing with offline classification and embedded robotic control (Figure S1 and Table S2). This architecture provides the conceptual basis for the following sections, in which the pressure–temperature sensor characteristics, synaptic plasticity, dual‐feature classification and robotic sorting are systematically investigated.

### 2.2 Design and Performance of the Integrated Iontronic Pressure–Temperature Sensor

To implement multimodal tactile reception in the iontronic‐transport afferent nerve, we designed an integrated pressure‐temperature sensor with a vertically stacked flexible architecture (Figure 2a). The device consists of a pressure‐sensitive iontronic layer, a temperature‐sensitive layer, patterned PI/Cu electrodes, a spacer, and a soft PDMS encapsulation layer. This multilayer configuration allows mechanical and thermal stimuli to be detected within a compact sensing unit while maintaining mechanical compliance for conformal contact with objects. The pressure‐sensitive component was constructed from a polyelectrolyte elastomer (PEE) containing ionic liquid species and a polymer network, in which mobile anions and cations redistribute under external mechanical loading [28]. The final formulation of PEE was determined through compositional regulation and systematic mechanical and electrical characterization using the replicate units and sample numbers specified for each test, with the key screening metrics reported as mean ± standard deviation, as shown in Figures S2–S10. The temperature‐sensitive layer was integrated underneath the pressure‐sensing unit to provide an additional thermal readout channel without increasing the lateral device footprint.

**FIGURE 2:**
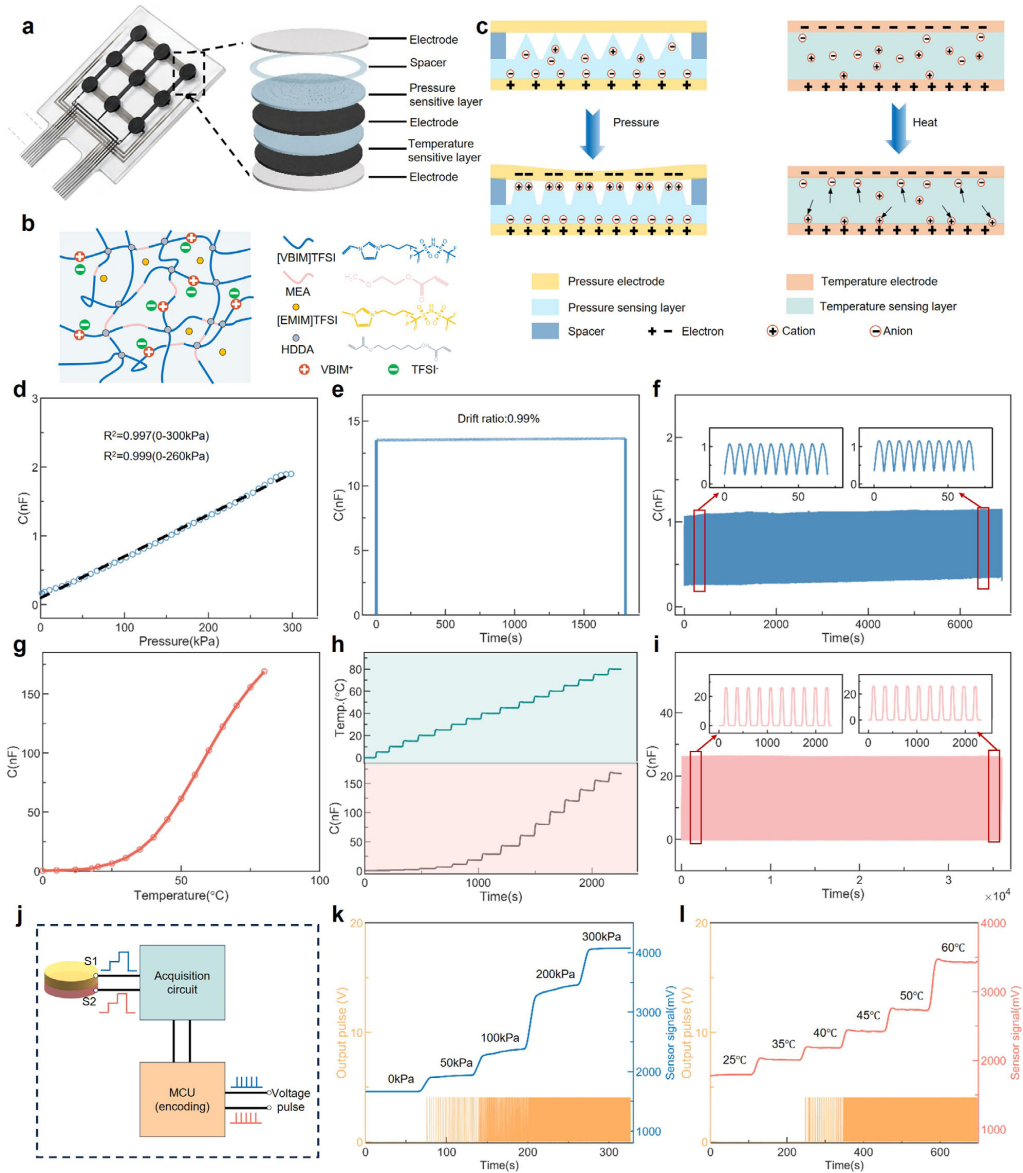
Structure, sensing mechanism, and electrical performance of the integrated pressure–temperature sensor. (a) Exploded view of the multilayer flexible sensor, consisting of a PDMS encapsulation layer, spacer, pressure‐sensitive layer, PI/Cu–PDMS electrode layers, and temperature‐sensitive layer. (b) Molecular and ionic composition of the iontronic sensing layer, including ionic liquid components and polymer network constituents. (c) Schematic illustration of the working mechanisms of the pressure and temperature sensing channels. Pressure changes the interfacial/contact state and ionic distribution in the pressure‐sensitive layer, whereas heating modulates ion/electron transport in the temperature‐sensitive layer. (d) Capacitance response of the pressure sensor under applied pressure, showing high linearity over 0–300 kPa and 0–260 kPa. (e) Long‐term signal stability of the pressure sensing channel, with a drift ratio of 0.99% at 80 kPa for 30 min, 25°C and 50% RH. (f) Cyclic pressure response showing stable and repeatable capacitance variation during repeated loading–unloading cycles. (g) Temperature‐dependent capacitance response of the temperature sensing channel. (h) Stepwise thermal response of the temperature sensor under increasing temperature. (i) Long‐term cyclic temperature response demonstrating signal repeatability and stability. (j) Schematic of the signal acquisition and pulse‐encoding circuit. (k) Sensor signal and corresponding encoded voltage pulses under stepwise pressure loading from 0 to 300 kPa. (l) Sensor signal and corresponding encoded voltage pulses under stepwise temperature variation from 25 to 60°C.

The sensing mechanism relies on stimulus‐dependent ion transport and interfacial capacitance modulation. Although both sensing layers employ the same PEE ionic material and share ion redistribution and electrical‐double‐layer (EDL) modulation as their underlying transduction processes, their modality selectivity arises primarily from their different structural transfer functions rather than from material composition alone. As shown in Figure 2c, under applied pressure, deformation of the pressure‐sensitive layer increases the effective contact area and alters the local ion distribution at the electrode interface, resulting in a pressure‐dependent capacitance variation [29]. This response is dominated by the pressure‐induced change in the effective EDL‐active contact area at the mechanically deformable microstructured interface. In contrast, the temperature‐sensitive layer maintains an approximately fixed interfacial geometry. Temperature variations primarily modulate the ionic conductivity of the PEE through changes in ion mobility, thereby affecting the EDL capacitance and producing a temperature‐dependent electrical response [30]. The corresponding sensing‐equation derivation is provided in Note S1. Owing to the separated functional layers, different structural transfer functions and independently addressed electrodes, the two outputs can be measured as separately addressed channels and distinguished after channel‐specific calibration, providing the hardware basis for dual‐modal tactile coding.

The pressure‐sensing channel exhibited a broad and highly linear response over the investigated pressure range (Figure 2d). The capacitance variation increased monotonically from 0 to 300 kPa, with a linear correlation coefficient of 0.997 [31], and showed even higher linearity from 0 to 260 kPa with a coefficient of 0.999, enabled by the linearly microstructured sensing layer [32] (Figure S14). Such linear behavior facilitates direct mapping of mechanical inputs into encoded pulse frequencies with minimal calibration complexity. The sensor also showed good long‐term operational stability, with a low drift ratio of only 0.99% during pressure monitoring at 80 kPa for 30 min, 25°C and 50% RH [33] (Figure 2e), which can be attributed to the low‐creep behavior and suppressed ion leakage of PEE [34] (Figures S10‐S12). Cyclic loading‐unloading tests further confirmed the repeatability of the pressure response, with stable signal profiles under repeated pressure stimuli (Figure 2f and Figure S15).

The temperature‐sensing channel exhibited a distinguishable but nonlinear response over the tested temperature range of 0–80°C (Figure 2g–i). The upward‐curving capacitance–temperature relationship over the full range was better described by a second‐order fit, whereas the first‐order expression in Equation S26 represents only a local approximation. Specifically, an approximately linear response was obtained over 25–45°C (R^2^ = 0.9705), which was therefore defined as the linear working range (Figure S23). Because the interfacial contact area of the temperature channel remained approximately fixed, the response primarily arose from the temperature dependence of the unit‐area EDL capacitance. Temperature‐dependent impedance spectra further showed that, at 1 kHz, |Z| decreased from 6.06 kΩ at 10°C to 1.41 kΩ at 60°C (Figure S24). To quantify residual reciprocal coupling under simultaneous thermo‐mechanical stimulation, we characterized both sensing channels over a 4 × 4 matrix comprising pressures of 0, 100, 200 and 300 kPa and temperatures of 10, 25, 45 and 60°C. The pressure‐channel output remained monotonically ordered with pressure at each tested temperature, whereas the temperature‐channel output remained monotonically ordered with temperature at each tested pressure (Figure S20a,b). However, the pressure channel retained appreciable temperature dependence and should therefore not be regarded as intrinsically or fully decoupled. We consequently used the measured temperature‐channel output as a reference variable in a two‐dimensional pressure–temperature calibration to compensate the pressure‐channel response. A related bimodal iontronic skin uses temperature‐referenced calibration of the pressure output [35]. The calibrated pressure results and quantified residual errors are presented in Figure S21c,d, with the calibration scope and error‐calculation procedures described in Note S6. The temperature channel showed limited pressure‐induced interference under coupled thermo‐mechanical stimulation (Figure S19).

We further evaluated the stability of each channel in separate measurements of the sensor under a constant pressure of 100 kPa and a temperature of 45°C for 60 min. The pressure‐ and temperature‐channel outputs exhibited relative drifts of 1.23% and 1.45%, respectively (Figure S22). The experimental procedure and drift definition are provided in Note S6. The integration of the two channels therefore enables pressure and temperature states to remain distinguishable after channel‐specific calibration, with quantified residual cross‐sensitivity. This capability allows the sensor to identify coupled tactile states, such as cold/soft and hot/hard objects, which cannot be reliably identified using a single sensing modality [36].

To minimize humidity‐induced changes in the hydration state and ion transport of the PEE, the integrated sensor was encapsulated with PDMS, and the effectiveness of this protective layer was evaluated under controlled humidity conditions. Temperature‐channel responses were measured from 25°C to 85°C, and pressure‐channel responses were measured from 0 to 200 kPa at 25°C, under RH levels of 50%, 70% and 90%. The unencapsulated devices exhibited pronounced RH‐dependent variations, whereas the PDMS‐encapsulated devices showed substantially reduced response divergence among the three humidity conditions (Figure S26; mean ± s.d., n = 3 repeated acquisitions).

Dynamic characterization was performed for both sensing channels. The pressure channel exhibited response and recovery times of 39.2 and 20.9 ms [37], respectively (Figure S16), characterizing the pressure‐sensor component robotic latency. The temperature‐channel heating–cooling trace is provided in Figure S18; a quantitative response/recovery time is not assigned because the archived timing information is inconsistent. When configured into a 3 × 3 array, the sensor exhibited highly consistent pressure and temperature responses across different pixels, confirming the uniformity and scalability of the dual‐modal tactile platform (Figures S27 and S28). The detailed calibration and pressure/temperature mapping procedures for the dual‐modal sensor array are described in Note S2 and Figure S29.

To connect the analog sensor outputs with the downstream artificial synaptic device, we developed a capacitance acquisition and spike‐encoding circuit (Figure 2j–l). The pressure and temperature signals were first acquired by the readout circuit and then converted by a microcontroller into voltage pulse trains. Increasing pressure from 0 to 300 kPa generated pressure‐dependent pulse sequences, while increasing temperature from 25°C to 60°C, covering most common tactile thermal conditions, produced temperature‐dependent encoded outputs. In this way, continuous physical stimuli were transformed into discrete neuromorphic spike trains, analogous to the conversion of receptor potentials into action‐potential sequences in biological afferent nerves [38]. The capacitance readout workflow, sampling grid, threshold window, pulse amplitude, pulse duration, inter‐pulse interval and capacitance‐to‐frequency mapping used for pressure and temperature encoding are summarized in Figure S30 and Table S7. These encoded pressure and temperature pulses serve as the presynaptic inputs for the artificial synaptic device, enabling subsequent neuromorphic processing and dual‐feature classification.

The signal‐acquisition and spike‐encoding subsystem consumed approximately 69.10 mW, of which the STM32 MCU system accounted for 66.20 mW (approximately 95.8%). The module‐level breakdown is provided in Table S8.

### 2.3 Synaptic Device Structure and Neuromorphic Plasticity Characteristics

To process the encoded tactile spike trains generated by the integrated pressure–temperature sensor, we fabricated an artificial synaptic device with an electrolyte‐gated nanowire transistor architecture [39] (Figure 3a). The device was constructed on a flexible PI substrate and consisted of a β‐SnO_2_ nanowire channel, Au source/drain electrodes, dual gate electrodes [40] and an ion‐gel dielectric layer composed of PVDF‐HFP and EMIM‐TFSI [41]. The SnO_2_ nanowire channel was selected for its compatibility with the electrolyte‐gated device architecture implemented here. Its gate‐controlled current modulation, pulse‐dependent accumulation, and post‐stimulation relaxation provide the history‐dependent electrical response required for tactile preprocessing, as demonstrated below. This configuration couples electronic transport in the semiconductor nanowire with ion redistribution in the electrolyte, providing a physical basis for emulating synaptic weight modulation [42] in biological afferent pathways. Inspired by ionic dynamics underlying signal transduction in biological neurons, the dual‐gate design further allows pressure‐ and temperature‐encoded spike inputs to be independently delivered to the synaptic device.

**FIGURE 3:**
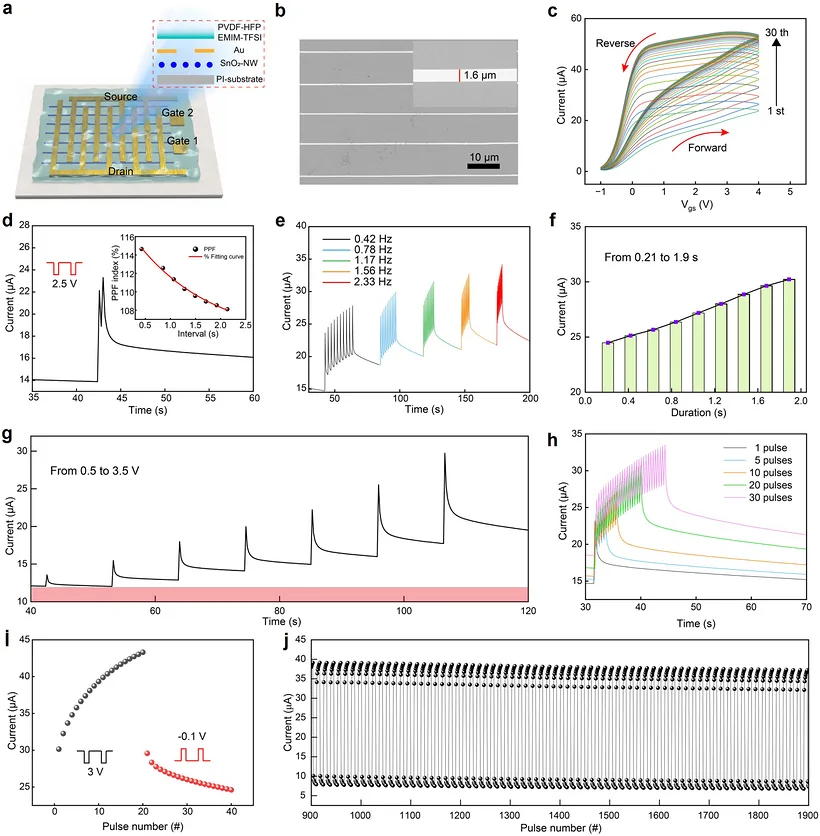
Synaptic device structure and neuromorphic plasticity characteristics. (a) Schematic of the flexible synaptic device based on a SnO_2_ nanowire channel, Au electrodes, PVDF‐HFP/EMIM‐TFSI electrolyte layer, and PI substrate. The device contains source, drain, and dual‐gate terminals for pulse modulation. (b) SEM image of the nanowire channel, with an enlarged view showing a channel feature size of approximately 1.6 µm. (c) Transfer characteristics of the synaptic device over 30 voltage sweeps, showing stable hysteretic modulation. (d) Paired‐pulse facilitation behavior triggered by two consecutive voltage pulses; the inset shows the paired‐pulse facilitation index as a function of pulse interval. (e) Frequency‐dependent postsynaptic current responses under pulse trains from 0.42 to 2.33 Hz. (f) Postsynaptic current as a function of pulse duration from 0.21 to 1.9 s. (g) Current modulation under different pulse amplitudes from 0.5 to 3.5 V. (h) Postsynaptic current responses to different pulse numbers, demonstrating pulse‐number‐dependent plasticity. (i) Bidirectional conductance modulation under excitatory and inhibitory pulse stimulation. (j) Cycling endurance of the synaptic device over repeated pulse stimulation, indicating reliable neuromorphic operation.

The morphology of the nanowire channel was characterized by scanning electron microscopy (Figure 3b). The channel region showed well‐defined parallel nanowire features with a characteristic spacing of approximately 1.6 µm, forming conductive pathways between the source and drain electrodes. When a gate voltage was applied, mobile ions in the ion‐gel layer migrated and accumulated at the electrolyte/semiconductor interface, forming an electric double layer [43]. This interfacial ion accumulation modulated carrier transport in the β‐SnO_2_ nanowire channel, resulting in a postsynaptic current response analogous to the excitatory postsynaptic current in biological synapses [44].

The basic gating behavior of the device was first evaluated by repeated transfer measurements (Figure 3c). During 30 consecutive forward and reverse gate‐voltage sweeps, the device exhibited pronounced hysteresis and gradual current modulation, indicating history‐dependent ion/electron coupling [45]. Such hysteretic behavior arises from the finite migration and relaxation dynamics of ions in the electrolyte, which allow the device conductance to retain information about previous electrical stimuli [46]. This property is essential for implementing short‐term memory and synaptic plasticity in the artificial afferent nerve [47].

We next investigated paired‐pulse facilitation, a representative form of short‐term synaptic plasticity [48]. When two consecutive presynaptic voltage pulses were applied, the second postsynaptic current peak was larger than the first one because residual ions from the first pulse had not fully relaxed before the arrival of the second pulse [49] (Figure 3d). The paired‐pulse facilitation index decreased as the pulse interval increased, consistent with the gradual dissipation of residual ionic polarization. This behavior closely resembles biological synapses, where the interval‐dependent accumulation and relaxation of neurotransmitter‐related effects determine the strength of the second postsynaptic response [50].

The synaptic device also displayed frequency‐dependent plasticity, which is critical for decoding spike‐frequency information from the upstream encoding circuit [5]. As the input pulse frequency increased from 0.42 to 2.33 Hz, the postsynaptic current progressively increased (Figure 3e). At higher frequencies, ions have insufficient time to return to their equilibrium distribution between adjacent pulses, leading to enhanced interfacial charge accumulation [51] and stronger channel modulation [52]. This frequency‐dependent response enables the artificial synapse to convert pressure‐ or temperature‐dependent pulse frequencies into distinguishable current states.

In addition to frequency dependence, the synaptic weight could be continuously regulated by pulse duration, amplitude and pulse number. Increasing the pulse duration from 0.21 to 1.9 s produced a monotonic enhancement of the postsynaptic current [53] (Figure 3f,g), reflecting pulse‐parameter‐dependent modulation of the electric‐double‐layer field. When the number of input pulses was increased from 1 to 30, the postsynaptic current showed cumulative enhancement followed by gradual relaxation after pulse removal (Figure 3h). These results demonstrate that the device can encode multiple spike parameters into analog conductance states, providing a versatile hardware platform for neuromorphic tactile feature extraction.

Bidirectional synaptic modulation was further achieved by applying pulses with opposite polarities. Positive gate pulses enhanced the postsynaptic current, whereas negative pulses suppressed the current [53] (Figure 3i). This excitatory/inhibitory behavior allows the synaptic device to emulate potentiation and depression, which are fundamental operations for neuromorphic learning and feature discrimination [54]. Finally, the cycling stability of the device was examined under repeated pulse stimulation (Figure 3j). The postsynaptic current maintained stable high‐ and low‐current states over a large number of switching cycles, confirming reliable operation under repeated neuromorphic stimulation [55].

To evaluate measurement repeatability, four measurements of the SnO_2_ nanowire synaptic transistor were collected under the same ten‐pulse stimulation protocol. All four measurements exhibited progressive PSC accumulation with increasing pulse number, while differences in response amplitude were observed (Figure S33a–c). The coefficient of variation of the baseline‐corrected pulse‐peak current ranged from 4.3% to 12.1% across the ten pulses, with an average of 8.2%. Following pulse cessation, exponential fits yielded relaxation time constants of 0.510, 0.530, 0.524, and 0.516 s, corresponding to a mean ± standard deviation of 0.520 ± 0.009 s and a coefficient of variation of 1.69%. The fitting R^2^ values ranged from 0.981 to 0.992 (Figure S33d). These repeated measurements do not establish device‐to‐device variability or long‐term retention.

Together, these results demonstrate that the artificial synaptic device provides physical temporal integration of the encoded tactile pulses. Ionic accumulation and relaxation cause the postsynaptic current to depend on both the incoming pulses and the residual state left by preceding stimulation, as evidenced by the paired‐pulse facilitation, frequency‐dependent response, and pulse accumulation and relaxation in Figure 3. The device therefore transforms discrete pulse sequences into history‐dependent current responses, which serve as the input to subsequent feature extraction and classification.

### 2.4 Dual‐Feature Neuromorphic Classification Based on Fused Pressure and Temperature Information

Inspired by the parallel processing of heterogeneous tactile cues in the biological somatosensory system, the pressure and temperature signals acquired from the integrated sensor were separately encoded into spike trains and delivered to the artificial synaptic device for feature extraction (Figure 4a). The resulting pressure‐ and temperature‐dependent postsynaptic current responses were then used as hardware‐generated neuromorphic features for classification.

**FIGURE 4:**
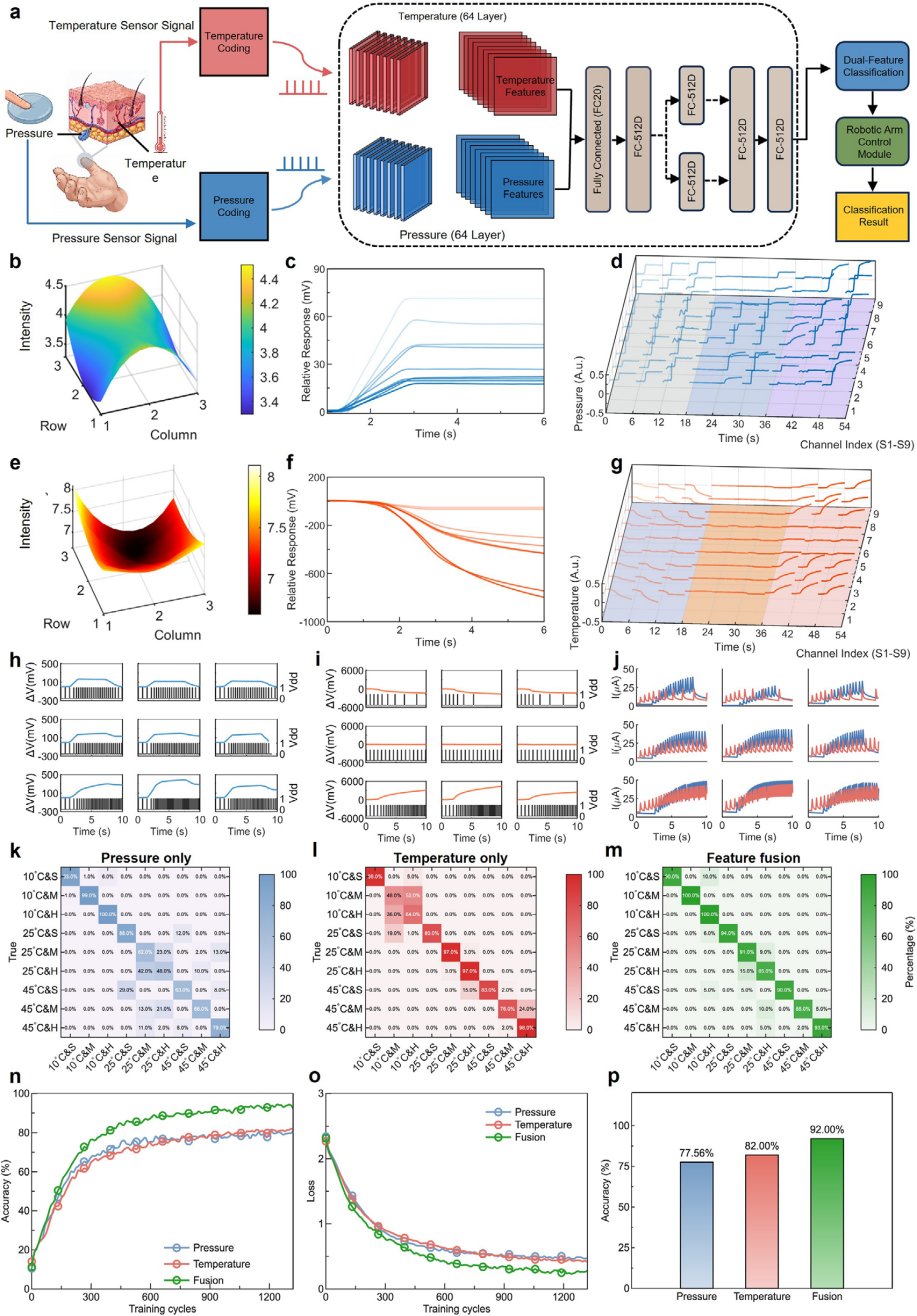
Dual‐feature neuromorphic classification based on fused pressure and temperature information. (a) Architecture of the dual‐modal classification system. Pressure and temperature sensor signals are separately encoded into pulse trains, processed through modality‐specific feature extraction, concatenated and classified by a common MLP for dual‐feature classification and robotic‐arm control. (b) Spatial pressure intensity distribution obtained from the sensor array. (c) Time‐dependent pressure response curves from different sensing channels. (d) Encoded pressure pulse patterns across multiple channels. (e) Spatial temperature intensity distribution obtained from the sensor array. (f) Time‐dependent temperature response curves from different sensing channels. (g) Encoded temperature pulse patterns across multiple channels. (h) Representative pressure‐coded spike trains under different pressure conditions. (i) Representative temperature‐coded spike trains under different temperature conditions. (j) Synaptic current responses generated by encoded pressure and temperature pulse inputs. (k) Confusion matrix of classification using pressure features only. (l) Confusion matrix of classification using temperature features only. (m) Confusion matrix of classification using fused pressure–temperature features. (n) Classification accuracy as a function of training cycles for pressure‐only, temperature‐only, and fusion‐based models. (o) Training loss evolution for the three classification strategies. (p) Comparison of final classification accuracy, showing that dual‐feature fusion achieves 92.00% accuracy, outperforming pressure‐only and temperature‐only recognition. Panels k–m show row‐normalized percentages under within‐recording contiguous‐block validation using the same fixed MLP. Pressure‐only and temperature‐only accuracies are 77.56% and 82.00%; fused macro‐F1 is 0.9215.

The classification architecture contains two independent input branches corresponding to the pressure and temperature channels. In each branch, the original sensor signal is first converted into a pulse sequence through the spike‐encoding circuit. These encoded pulses are applied to the artificial synaptic device, where the frequency‐, amplitude‐ and history‐dependent ionic dynamics transform the spike inputs into analog postsynaptic current states. The pressure and temperature features are then concatenated before entering a common fully connected multilayer perceptron (MLP) for dual‐feature classification [56]. This neuromorphic preprocessing strategy transforms tactile spike inputs into history‐dependent synaptic states for numerical feature extraction [57].

To visualize the spatial distribution of external stimuli, we first recorded pressure and temperature mappings from the sensor array under representative tactile conditions (Figure 4b,e). The use of a 3 × 3 array is essential here because it allows the tactile input to be represented as a spatially resolved feature map rather than as a single pressure or temperature value. In contrast, a single pressure sensor combined with a single temperature sensor would only provide two global scalar signals and would not resolve the contact location, loading distribution, or local thermal contrast across the sensing area. The 3 × 3 array responses were then converted into one‐dimensional feature vectors using row‐wise vectorization, an order‐preserving flattening procedure in which the nine pixel outputs were sequentially arranged from the first row to the third row (Note S3). This vectorization preserves the relative pixel positions within the array, enabling the neuromorphic classifier to use both stimulus intensity and spatial distribution as input features. The pressure channel generated distinguishable spatial intensity patterns corresponding to the mechanical loading region, whereas the temperature channel provided thermal contrast associated with the local temperature distribution. The time‐dependent responses from multiple sensing units further confirmed that pressure and temperature signals could be independently acquired from the integrated array (Figure 4c,f). After pulse encoding, these analog signals were converted into discrete spike trains with modality‐dependent temporal patterns (Figure 4d,g). Higher pressure or temperature inputs produced denser pulse sequences, enabling stimulus intensity to be represented in the temporal domain. Therefore, the array serves not merely as a collection of pressure and temperature sensors, but as a spatial encoding interface that converts coupled mechanical–thermal stimuli into multimodal feature maps for subsequent neuromorphic fusion and classification. At this scale, row‐wise vectorization preserves the relative pixel positions while maintaining a compact feature representation. Extension to larger arrays will require coordinated optimization of array addressing, multiplexed readout and pulse delivery, together with compensation for synaptic‐device variability and control of the increasing classifier dimensionality.

The encoded pressure and temperature pulses were then used to stimulate the artificial synaptic device. Owing to the synaptic plasticity characterized in Section 2.3, different pulse patterns generated distinguishable postsynaptic current responses (Figure 4h–j). Pressure‐coded inputs mainly reflected mechanical features such as contact force and hardness, whereas temperature‐coded inputs represented thermal features. These two feature sets are complementary: pressure information can distinguish soft and hard objects, while temperature information can distinguish cold and hot objects [58]. Therefore, combining both modalities is expected to resolve ambiguous object states that cannot be reliably separated using either pressure or temperature alone.

For reproducible classification, the hardware‐generated postsynaptic‐current traces were further converted into numerical feature vectors before model training. Each pressure or temperature branch retained channel‐specific PSC descriptors, including local current‐window values, derivative‐window values, instantaneous PSC values and integrated PSC‐area features. The features were normalized using training‐set statistics and then supplied to a common multilayer perceptron, where the pressure and temperature branches were fused only after modality‐specific feature extraction. The complete input dimensions, PSC‐feature definitions, train/test split, normalization procedure, model hyperparameters and random‐seed settings are provided in Note S4 and Table S9.

We compared three classification strategies: pressure‐only classification, temperature‐only classification and dual‐feature fusion. When only pressure features were used, the model could separate objects with different mechanical properties but showed ambiguity between samples with similar hardness and different temperatures (Figure 4k). Conversely, temperature‐only classification distinguished thermally different samples but showed limited ability to resolve objects with similar temperatures and different mechanical properties (Figure 4l). In contrast, the fused pressure–temperature model produced a more diagonal confusion matrix with fewer misclassified samples, demonstrating that the two sensory modalities provide complementary discriminative information [59] (Figure 4m).

The superiority of dual‐feature fusion was further verified by the training accuracy and loss curves (Figure 4n,o). During training, the fusion‐based model converged more rapidly and reached a higher final accuracy than either single‐modal model. The final comparison showed that dual‐feature neuromorphic classification achieved an accuracy of 92.00%, under the within‐recording contiguous‐block protocol, outperforming both pressure‐only and temperature‐only strategies under the tested conditions [5] (Figure 4p). These results demonstrate that the iontronic‐transport afferent nerve can not only encode pressure and temperature signals into neuromorphic spike patterns, but also use synaptic plasticity to extract separable features for high‐accuracy tactile classification. To provide the supporting metrics, the fused confusion‐matrix values, accuracy summary and per‐class precision, recall and macro‐F1 scores are further provided in Figure S34 and Table S10. These additional metrics confirm that the improvement is not limited to the overall accuracy value but is also reflected in balanced class‐level recognition performance.

To assess the contribution of synaptic preprocessing, we compared encoded‐pulse features with PSC‐derived features. Both representations were evaluated using the same class/frame‐index pairing, within‐recording contiguous‐block partition, MLP classifier settings, and evaluation metrics. For fused pressure–temperature classification, the encoded‐pulse baseline achieved an accuracy of 53.44%, whereas the PSC‐based representation achieved 92.00%, corresponding to an increase of 38.56 percentage points. These results support the benefit of the history‐dependent PSC representation for classification under the tested conditions. The single‐modal comparisons and confusion matrices are provided in Figure S31, with the evaluation protocol and performance metrics detailed in Note S4 and Table S10.

Overall, this dual‐feature classification framework establishes the functional link between multimodal tactile sensing and robotic perception. By integrating pressure and temperature coding with artificial synaptic processing, the system converts external physical stimuli into discriminative neuromorphic features. This capability provides the basis for the robotic sorting demonstration described in the next section, where objects with coupled color, temperature and stiffness attributes are identified and manipulated through the integrated artificial afferent system.

### 2.5 Neuromorphic Robotic Sorting Based on Color, Temperature, and Stiffness Perception

To demonstrate the practical utility of the iontronic‐transport afferent nerve in embodied robotic perception, we further integrated the pressure–temperature sensor, spike‐encoding circuit, artificial synaptic device and robotic control module into a neuromorphic sorting system (Figure 5a). In this task, objects were designed to possess coupled visual, thermal and mechanical attributes, including different colors and stiffness levels. Instead of using an optical camera, color information was indirectly converted into thermal contrast through a photothermal process and then detected by the temperature‐sensing channel [60]. Meanwhile, object stiffness was detected by the pressure‐sensing channel under a fixed displacement. This strategy allowed the robotic system to recognize object states using non‐visual tactile and thermal information.

**FIGURE 5:**
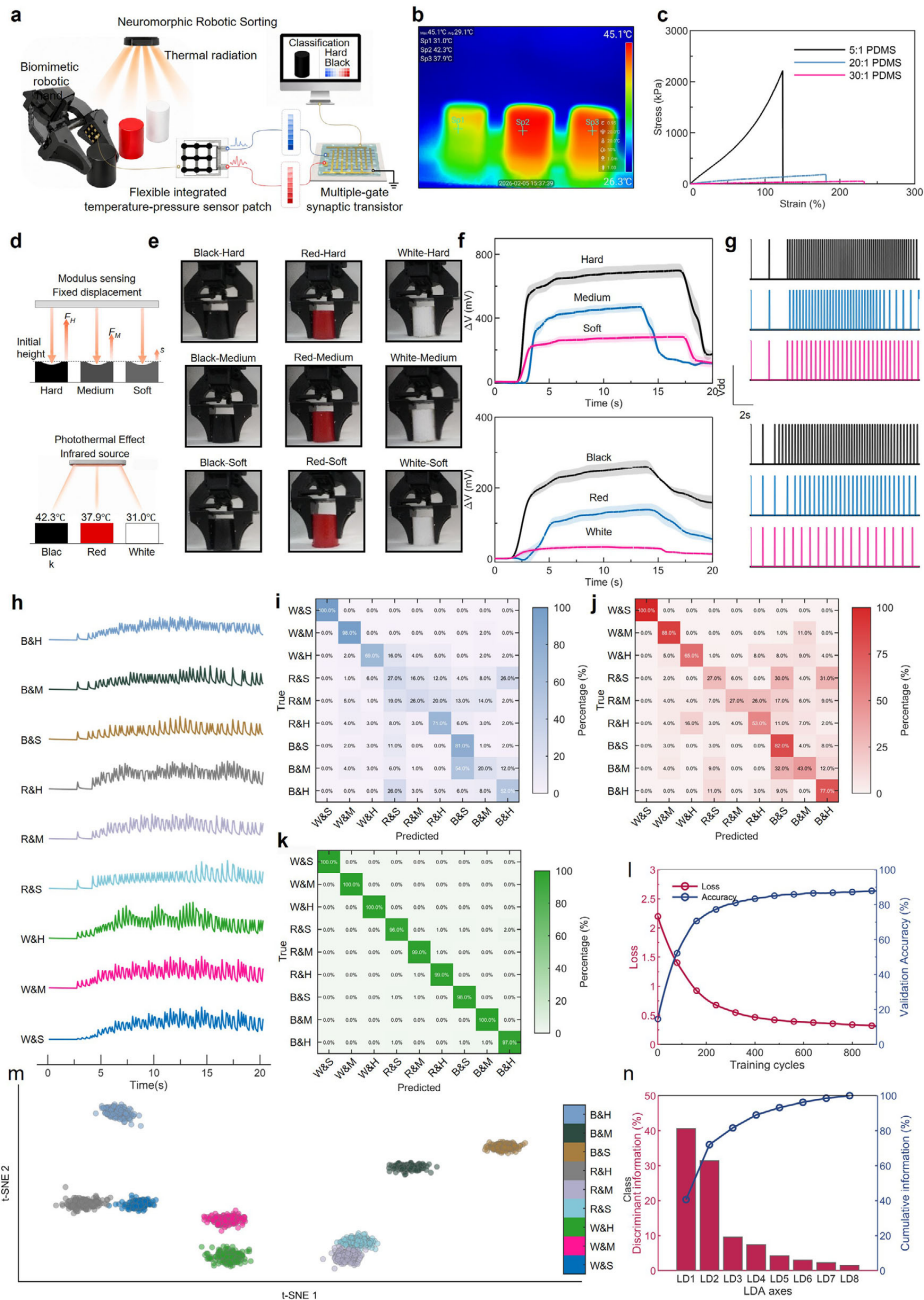
Neuromorphic robotic sorting based on color, temperature, and stiffness perception. (a) Schematic illustration of the robotic sorting system based on a flexible integrated pressure–temperature sensor patch and offline neuromorphic classification workflow. The system detects thermal radiation and mechanical stiffness from objects and outputs classification results for robotic control. (b) Infrared thermal image of black, red, and white cylindrical objects, showing color‐dependent photothermal temperature differences under illumination. (c) Stress–strain curves of PDMS samples with different mixing ratios, corresponding to different stiffness levels. (d) Sensing principles for distinguishing object stiffness through mechanical displacement and object color through photothermal temperature differences. (e) Photographs of nine test objects with different combinations of color and stiffness, including black, red, and white objects with hard, medium, and soft mechanical properties. (f) Sensor voltage responses to objects with different stiffness and photothermal characteristics. (g) Encoded pulse trains generated from the sensor responses for neuromorphic processing. (h) Synaptic current responses corresponding to nine combined object states, labeled as T1H1 to T3H3. (i) Confusion matrix based on single‐modal mechanical or pressure‐related features. (j) Confusion matrix based on single‐modal thermal or color‐related features. (k) Confusion matrix based on fused dual‐feature recognition, showing improved classification performance. (l) Training loss and validation accuracy during neuromorphic classification. (m) t‐SNE visualization of the learned feature distribution, showing separable clusters for the nine object categories. (n) Linear discriminant analysis of feature contribution, indicating the major discriminative axes used for object classification. T1/T2/T3 correspond to white/red/black and H1/H2/H3 to soft/medium/hard.

Three colors—black, red and white—were selected to produce different photothermal responses under infrared irradiation. Infrared thermal imaging showed that the objects reached distinguishable surface temperatures after illumination, with darker objects exhibiting higher temperature owing to stronger light absorption [61] (Figure 5b). In parallel, three stiffness levels were prepared using PDMS samples with different mixing ratios. The stress–strain curves confirmed that the 5:1 PDMS sample showed the highest modulus and was assigned as the hard sample [62], whereas the 20:1 and 30:1 PDMS samples corresponded to medium and soft samples, respectively (Figure 5c). By combining three color‐derived thermal states with three stiffness states, nine object categories were constructed, including black‐hard, black‐medium, black‐soft, red‐hard, red‐medium, red‐soft, white‐hard, white‐medium and white‐soft samples (Figure 5e).

The sensing principles for the two modalities are illustrated in Figure 5d. Under a fixed mechanical displacement, harder samples generate larger reaction forces and therefore stronger pressure‐sensor outputs, whereas softer samples deform more readily and produce lower pressure responses. At the same time, the photothermal temperature difference generated by infrared irradiation provides an independent thermal feature associated with object color. Thus, each object can be represented by a combined pressure–temperature signature, where stiffness is encoded in the pressure channel and color‐related thermal absorption is encoded in the temperature channel.

The integrated sensor produced distinguishable voltage responses for the different object states (Figure 5f). The pressure‐related responses separated hard, medium and soft samples according to their mechanical stiffness, while the temperature‐related responses distinguished black, red and white samples according to their photothermal heating levels. These analog signals were then converted into pulse trains by the spike‐encoding circuit (Figure 5g). Objects with higher pressure or temperature responses generated denser pulse sequences, allowing both mechanical and thermal stimulus intensities to be represented in the temporal domain. The encoded spike trains were subsequently applied to the artificial synaptic device, producing postsynaptic current responses with distinct temporal profiles for the nine temperature–hardness combinations, labeled T1H1 to T3H3 (Figure 5h). The full correspondence between the T1H1–T3H3 labels and the black/red/white, hard/medium/soft object states, together with representative voltage, encoded‐pulse and synaptic‐current waveforms for the nine classes, is provided in Figure S32. T1, T2 and T3 denote white, red and black objects, respectively; H1, H2 and H3 denote soft, medium and hard states.

We next evaluated the classification performance of single‐modal and dual‐modal neuromorphic recognition. When only pressure‐derived features were used, the system could primarily distinguish hardness levels but showed substantial confusion among objects with similar stiffness and different colors (Figure 5i). Conversely, when only temperature‐derived features were used, the model separated color‐induced thermal states more effectively but exhibited ambiguity among objects with similar photothermal responses and different stiffness levels (Figure 5j). In contrast, the fused pressure–temperature classification produced a strongly diagonal confusion matrix, indicating accurate discrimination of all nine object categories (Figure 5k). The training curves further showed a continuous decrease in loss and an increase in validation accuracy with increasing training cycles, confirming stable convergence of the neuromorphic classifier (Figure 5l).

The separability of the learned dual‐modal features was further visualized using dimensionality‐reduction analysis. The t‐SNE projection showed that the nine object categories formed well‐separated clusters in the feature space [63], demonstrating that the pressure‐temperature fusion strategy generated discriminative representations for robotic sorting (Figure 5m). Linear discriminant analysis revealed that the first few discriminant axes contributed most of the classification information [64], suggesting that the fused neuromorphic features can be efficiently compressed while retaining high discriminative capability (Figure 5n). The dimensionality‐reduction inputs and analysis settings are summarized in Table S11.

Finally, the classification output was used to guide robotic sorting. After the flexible sensor patch contacted an object, the pressure and temperature signals were acquired, encoded into spike trains and processed by the artificial synaptic device. The offline classification result was then sent to the robotic arm control module, enabling the robot to identify and sort objects according to their combined color‐derived thermal state and mechanical stiffness, as demonstrated in Movie S1. The feed‐forward signal flow from fixed‐displacement contact to robotic actuation is summarized in Note S5. This demonstration shows that the iontronic‐transport afferent nerve supports feed‐forward tactile perception and robotic decision‐making, providing a hybrid hardware–software route toward multimodal artificial skin for intelligent robotic manipulation [65].

## 3 Conclusions

In summary, we have demonstrated an iontronic‐transport artificial afferent nerve that couples multimodal tactile sensing, spike encoding, synaptic plasticity, and robotic decision‐making in an integrated hybrid hardware–software pathway. The PEE‐based pressure‐temperature sensor provides a broad and highly linear pressure response, stable thermal response, low signal drift, fast dynamic behavior, and reproducible array outputs. Through capacitance acquisition and microcontroller‐based encoding, pressure and temperature signals are converted into stimulus‐dependent pulse trains that directly drive an electrolyte‐gated artificial synaptic device. The synaptic device emulates key plasticity functions, including paired‐pulse facilitation, frequency‐dependent postsynaptic‐current modulation, pulse‐duration and pulse‐amplitude dependence, bidirectional conductance regulation, and cycling stability. By combining these hardware‐generated features with dual‐feature classification, the system improves recognition under the tested temporal‐block protocol and supports feed‐forward robotic sorting of objects with different thermal and mechanical attributes. This integrated strategy offers a promising platform for flexible artificial skin, near‐sensor tactile computing, and intelligent robotic manipulation, while future work should focus on experimentally benchmarking synchronized contact‐to‐decision latency and complete energy per decision, including synaptic stimulation, PSC acquisition, embedded classification, and command generation. Scaling beyond the present 3 × 3 prototype will additionally require multiplexed or active‐matrix addressing, parallel pulse routing, lower‐power embedded processing, compensation for synaptic‐device variability, and validation under long‐term and more complex real‐world contact conditions.

## 4 Experimental Section

### 4.1 Materials

Vinyl‐3‐butylimidazolium bis(trifluoromethylsulfonyl)imide([VBIM][TFSI], 3300261E), 1‐ethyl‐3‐methylimidazolium bis[(trifluoromethyl)sulfonyl]imide ([EMIM][TFSI], 30306A), ethylene glycol monomethyl ether acrylate (MEA, E808702), and phenylbis(2,4,6‐trimethylbenzoyl)phosphine oxide (Irgacure 819, 104359B) were purchased from Adamas. 1,6‐Hexanediol diacrylate (HDDA, H102721) was purchased from Aladdin. Ecoflex‐30 was purchased from Smooth‐On. PDMS films were purchased from Hangzhou Weisichuang Technology Co., Ltd. PI/Cu films and double‐sided adhesive spacers were commercially obtained and used as received unless otherwise specified.

### 4.2 Synthesis of Polyelectrolyte Elastomer

To synthesize the polyelectrolyte elastomer (PEE), 1 g [VBIM][TFSI], 0.604 g MEA, 0.2 g [EMIM][TFSI], 0.037 g HDDA, and 0.01 g Irgacure 819 were mixed. After being magnetically stirred for 1 h at room temperature, the obtained homogeneous PEE precursor solution was poured onto the secondary Ecoflex template and cured under 405 nm ultraviolet light (30 W) for 1 h. Finally, the cured PEE film was carefully peeled off from the Ecoflex template for subsequent sensor fabrication.

### 4.3 Fabrication of the Bimodal Iontronic Sensor

#### 4.3.1 Template Fabrication

The microstructured template was first designed and modeled using SolidWorks. The original high‐precision resin template was then sliced and printed using a commercial BMF S230 3D printer with an optical accuracy of 2 µm. After printing, the resin template was immersed in absolute ethanol for cleaning and further exposed to UV light (30 W) for 24 h to complete post‐curing. Subsequently, the original resin template was placed in a Petri dish, and Ecoflex‐30 precursor with a mass ratio of 1:1 was poured onto the template. After degassing under vacuum for 1 min, the Ecoflex‐30 was cured at room temperature for 3 h. Finally, the secondary Ecoflex template with the inverse microstructure was carefully peeled off from the original resin template.

#### 4.3.2 Fabrication of the Pressure‐Sensing Module

The microstructured PEE pressure‐sensitive film was prepared by casting 30 µL of the as‐prepared PEE precursor solution onto the secondary Ecoflex template. The precursor was degassed under vacuum for 1 min to remove trapped air bubbles and then photopolymerized under 405 nm UV light (30 W) for 1 h. After curing, the microstructured PEE film was carefully peeled off from the Ecoflex template. The pressure‐sensing module was then assembled by sequentially stacking the upper PI/Cu electrode, the spacer, the microstructured PEE sensing film, and the lower PI/Cu electrode. The obtained pressure‐sensing module was stored for subsequent integration.

#### 4.3.3 Fabrication of the Temperature‐Sensing Module

To fabricate the temperature‐sensing module, an upper PI/Cu electrode was first placed on a glass slide. A 100 µm‐thick adhesive PET film with a matched geometry was patterned using a laser cutter and attached around the electrode region to form a 100 µm‐thick recessed cavity. Then, 30 µL of the as‐prepared PEE precursor solution was dropped into the cavity. A release PET film was placed on top to flatten the precursor droplet, followed by photopolymerization under 405 nm UV light (30 W) for 1 h. After curing, the lower PI/Cu electrode was attached onto the cured PEE layer to obtain the temperature‐sensing module.

#### 4.3.4 Assembly and Encapsulation of the Bimodal Sensor

The pressure‐ and temperature‐sensing modules were vertically integrated with a PDMS film inserted between them as an isolation layer. Finally, the entire device was encapsulated using PDMS films treated by oxygen plasma at 60 W for 1 min. The plasma‐treated PDMS encapsulation improved interfacial bonding, structural integrity, and mechanical robustness of the bimodal iontronic sensor.

#### 4.3.5 Characterization of Iontronic Sensors

The capacitance signals of the iontronic sensors were recorded using an LCR digital bridge (TH2840) in the Cs–Rs mode. Unless otherwise specified, the testing frequency was set to 10 kHz, with an AC voltage of 1 V, a DC bias of 100 mV, and a medium scanning speed.

For the pressure‐capacitance calibration, the pressure‐sensing module was loaded using a motorized force tester (MARK‐10) over a pressure range of 0–300 kPa. The loading rate was 1 mm min^−^^1^, and the mechanical data were recorded at 10 points s^−^^1^. Capacitance was simultaneously measured using a TH2840 LCR meter in the Cs–Rs mode at 10 kHz, with an AC excitation voltage of 1 V and a DC bias of 100 mV. The cyclic compression test was conducted separately at 15 mm min^−^^1^ for 1000 loading–unloading cycles.

For the sustained‐load drift test, a constant pressure of 80 kPa was applied for 30 min at 25°C and 50% RH. The capacitance was continuously recorded throughout the loading period, and the drift ratio was calculated as |C(t) − C_0_|/C_0_ × 100%, where C_0_ is the capacitance at the beginning of the holding period.

For the cyclic compression test, repeated loading–unloading cycles were applied using the MARK‐10 force tester. The pressure range was set to 0–300 kPa, the loading speed was 15 mm min^−^^1^, and the number of cycles was 1000. The mechanical data were recorded at a sampling rate of 10 points s^−^^1^, while the capacitance response was simultaneously collected using the LCR digital bridge.

The temperature response of the temperature‐sensing module was characterized using a temperature‐controlled stage (Mk2000B). For the temperature–capacitance response measurement, the temperature was varied from 0°C to 80°C under the Hold mode, and the corresponding capacitance signal was recorded using the LCR digital bridge. For the stepwise temperature response test, the temperature was increased from 0°C to 80°C with an interval of 10°C, and each temperature plateau was maintained for 10 min. The capacitance response at each plateau was continuously recorded.

For the thermal cycling test, the temperature‐sensing module was repeatedly heated and cooled between 0°C and 80°C for 1000 cycles using the temperature‐controlled stage under the Hold mode. The capacitance signal was continuously monitored using the LCR digital bridge throughout the cycling test.

### 4.4 Sensor Array Mapping and Decoupling Tests

For array‐level measurements, the pressure‐ and temperature‐sensing units were addressed independently through the corresponding electrode channels. Spatial pressure and temperature maps were reconstructed from the calibrated capacitance responses of individual pixels. To evaluate thermo‐mechanical decoupling, the temperature‐sensing channel was monitored under increasing external pressure, and the pressure‐induced error was calculated from the deviation of the temperature‐channel output relative to the pressure‐free baseline. Joint calibration was additionally performed over pressures of 0, 100, 200 and 300 kPa and temperatures of 10, 25, 45 and 60°C, with three repeated acquisitions per condition; these repeats were not treated as independent‐device measurements (Figures S20 and S21).

### 4.5 Spike‐Encoding Circuit and Pulse Generation

The capacitance response of the pressure and temperature channels was acquired by the capacitance readout circuit and transmitted to the microcontroller unit. The microcontroller converted the analog sensor signals into voltage pulse trains according to the calibrated stimulus‐output relationship. Higher pressure or temperature responses were encoded as denser pulse sequences, which were used as presynaptic inputs for the artificial synaptic device. The encoded pressure and temperature pulses were generated separately to preserve modality‐specific temporal information before feature fusion.

### 4.6 Artificial Synaptic Device Fabrication and Electrical Characterization

The artificial synaptic device was constructed on a flexible PI substrate using Au source/drain electrodes, dual gate electrodes, a β‐SnO_2_ nanowire channel, and a PVDF‐HFP/EMIM‐TFSI ion‐gel dielectric. Gate‐voltage sweeps were used to evaluate the transfer characteristics and hysteresis behavior. Presynaptic voltage pulses with controlled interval, frequency, duration, amplitude, polarity, and pulse number were applied to the gate terminals, and the drain current was recorded as the postsynaptic current. Paired‐pulse facilitation, frequency‐dependent plasticity, pulse‐parameter‐dependent modulation, excitatory/inhibitory regulation, relaxation behavior, and cycling endurance were analyzed from the resulting current responses.

### 4.7 Neuromorphic Feature Extraction and Classification

Pressure and temperature sensor outputs were first transformed into encoded pulse trains and then applied to the artificial synaptic device to obtain postsynaptic‐current features. For the dual‐feature classification task, pressure‐derived and temperature‐derived features were processed channel‐wise and concatenated before a common MLP classifier. Pressure‐only, temperature‐only and fused pressure–temperature strategies were compared using the same temporal‐block protocol. The Figure 4 dataset comprised nine continuous class records with 100 frames per class; these frames were not independent trials. Pulse and measured single‐channel PSC representations were matched by class and frame index, and cumulative descriptors were computed before partitioning. The separate 27‐record offline analysis associated with Figure 5 is described in Note S7. Feature definitions, input dimensions, data partitions, normalization procedures and model parameters are detailed in Note S4 and Table S9.

Four measurements of the SnO_2_ nanowire synaptic transistor were collected using an identical train of ten pulses. Drain‐current traces were baseline‐aligned before averaging. The peak current associated with each pulse was extracted for each measurement, and the mean and sample standard deviation were calculated across the four measurements. Post‐stimulation relaxation was fitted using a single‐exponential model to obtain the relaxation time constant for each measurement. The coefficient of variation was calculated as the sample standard deviation divided by the mean, multiplied by 100%. The corresponding PSC traces and relaxation fits are shown in Figure S33.

### 4.8 Robotic Sorting Demonstration

Cylindrical objects with three colors (black, red, and white) and three stiffness levels were prepared to construct nine object categories. The stiffness levels were obtained using PDMS samples with different base/curing‐agent ratios, and the color‐dependent thermal states were generated by infrared illumination through photothermal conversion. During sorting, the flexible sensor patch contacted each object under a fixed displacement. The pressure and temperature responses were acquired, encoded into pulse trains, processed by the synaptic device, and classified offline by the dual‐feature recognition model. The classification result was transmitted to the robotic control module to trigger the corresponding sorting action.

### 4.9 Statistical Analysis and Data Processing

Unless otherwise stated, calibration and repeatability tests were performed using independently acquired measurement cycles. Linear fitting was used to evaluate the pressure‐response linearity, and the coefficient of determination (R^2^) was calculated over the specified pressure range. Drift ratio was calculated from the relative capacitance variation during sustained loading. Classification metrics were calculated from the corresponding confusion matrices, and dimensionality‐reduction analyses were performed using t‐SNE and linear discriminant analysis to visualize feature separability. The abbreviations, supporting tables and figure‐to‐SI mappings are provided in Tables S1, S4 and S6.

The device geometry, layer thicknesses, electrode dimensions, array configuration, measurement conditions and spike‐encoding parameters are consolidated in Tables S5 and S7.

## Author Contributions

T.P. and J.G. conceptualized the idea, directed the study and supported the project. M.D., W.C., J.G., and X.G. fabricated and characterized the devices, as well as performed the experiments. H.X. and F.W. fabricated the iontronic electronic sensing composites. M.D., W.C. and W.W. analyzed the data. C.D. and Z.L. provided valuable suggestions and insights throughout the development of the manuscript. T.P., W.X., and J.G. revised the manuscript. All authors provided constructive and valuable feedback on the paper.

## Conflicts of Interest

The authors declare no conflicts of interest.

## Supporting information




**Supporting File 1**: advs78621‐sup‐0001‐SuppMat.docx.

**Supporting File 2**: advs78621‐sup‐0002‐MovieS1.MOV.

## Data Availability

The data that support the findings of this study are available from the corresponding author upon reasonable request.
