# Supplementary material for: An Iontronic Bimodal Afferent Nerve for Cross‐Modal Neuromorphic Fusion and Robotic Sorting
Source: Adv Sci (Weinh). 2026 Nov 10:e78621. Online ahead of print. doi: 10.1002/advs.78621 (PMC13752246; doi:10.1002/advs.78621)
Supplement: Supplementary file 1 [file ADVS-9999-e78621-s001.docx]

Supplementary Materials for

**An Iontronic Bimodal Afferent Nerve for Cross-Modal Neuromorphic Fusion and Robotic Sorting**

*Mengkang Deng^1,2†^, Wenxin Cao^1,2†^, Xinci Gong^1,2†^, Jiangdong Gong^1,2,5†,*^, Hang Xu^1,2^, Wenjing Wang^2,4^, Chentao Du^1,2,3^, Fei Wang^1,2^, Zhiguang Liu^4^, Wentao Xu^6,*^ and Tingrui Pan^1,2,4,*^*

^1^School of Biomedical Engineering, University of Science and Technology of China, Hefei 230026, China.

^2^Center for Intelligent Medical Equipment and Devices, Suzhou Institute for Advanced Research, University of Science and Technology of China, Suzhou 215123, China

^3^Department of Biomedical Engineering, City University of Hong Kong, Hong Kong 999077, China.

^4^Department of Precision Machinery and Instrumentation, University of Science and Technology of China, Hefei 230026, China

^5^Department of Applied Biology and Chemical Technology and Research Institute for Smart Energy, The Hong Kong Polytechnic University, Hung Hom, Kowloon, Hong Kong 999077, China

^6^Institute of Photoelectronic Thin Film Devices and Technology, Key Laboratory of Photoelectronic Thin Film Devices and Technology of Tianjin, College of Electronic Information and Optical Engineering, Nankai University, Tianjin 300350, China

*Corresponding author: jiangdong.gong@polyu.edu.hk, wentao@nankai.edu.cn, tingrui@ustc.edu.cn.

†These authors contributed equally to this work.

**This PDF file includes:**

Supplementary Notes 1–7

Supplementary Figures S1–S34

Supplementary Tables S1–S11

Supplementary References

**Other Supplementary Materials for this manuscript include the following:**

Supplementary Movies S1

**Supplementary Notes**

**Supplementary Note 1. Sensing-equation derivation for pressure and temperature channels**

The pressure-sensing channel is treated as an iontronic interfacial capacitor, whose capacitance originates from the electrical double layer (EDL) formed at the electrode/PEE interface. According to the Gouy–Chapman–Stern model, the EDL consists of a compact Helmholtz layer and a diffuse ionic layer^1,2^. These two layers can be electrically described as two capacitive components connected in series, namely the Helmholtz capacitance $C_{H}$ and the diffuse-layer capacitance $C_{D}$.

$$\begin{aligned} C_{\text{EDL}}=\left( \frac{1}{C_{\text{H}}}+\frac{1}{C_{\text{D}}} \right)^{-1}\#\left( S1 \right) \end{aligned}$$

For a given electrode/PEE interface, $C_{H}$ and $C_{D}$ are both proportional to the effective interfacial contact area $A$. Therefore, the EDL capacitance can be expressed as

$$\begin{aligned} C_{\text{EDL}}=\eta_{A}\cdot\phi\left( d,\varepsilon,c,\Phi,T \right)\cdot A=\text{UAC}\cdot A\#\left( S2 \right) \end{aligned}$$

where $\eta_{A}$ is the interfacial roughness factor, $d$ is the effective Helmholtz-layer thickness, $\varepsilon$ is the dielectric constant of the ion-containing medium, $c$ is the ion concentration, $\Phi$ is the interfacial potential, and $T$ is the temperature. The term $\phi\left( d,\varepsilon,c,\Phi,T \right)$ summarizes the physicochemical contribution of the electrode/PEE interface to EDL formation. Thus,

$$\begin{aligned} \text{UAC}=\eta_{A}\cdot\phi\left( d,\varepsilon,c,\Phi,T \right)\#\left( S3 \right) \end{aligned}$$

is defined as the unit-area capacitance of the iontronic interface.

Under the small-signal capacitance measurement used in this work, the physicochemical parameters $d,\varepsilon,c,\Phi,$ and $T$ are assumed to remain approximately constant within the calibrated pressure range. Therefore, the pressure-induced capacitance variation is mainly governed by the change in the effective contact area $A$^3,4^, rather than by a change in the intrinsic $UAC$. Equation (S2) can therefore be rewritten as

$$\begin{aligned} C_{\text{EDL}}\left( P \right)=\text{UAC}\cdot A\left( P \right)\#\left( S4 \right) \end{aligned}$$

where $A\left( P \right)$ is the pressure-dependent effective electrode/PEE contact area.

For the present sandwich-structured iontronic pressure sensor, the capacitance change is proportional to the increase in pressure. The PEE layer forms two EDL interfaces with the upper and lower electrodes. The upper electrode/PEE interface is pressure-sensitive because the deformation of the microstructured PEE layer progressively increases the effective contact area under normal loading. In contrast, the lower electrode/PEE interface is assumed to be stably attached, and its contact area remains nearly unchanged during loading.

Thus, the upper and lower interfacial EDL capacitances can be written as

$$\begin{aligned} C_{\text{top}}\left( P \right)=\eta_{A,\text{top}}\phi_{\text{top}}\left( d,\varepsilon,c,\Phi,T \right)A_{\text{top}}\left( P \right)=\text{UAC}_{\text{top}}A_{\text{top}}\left( P \right)\#\left( S5 \right) \end{aligned}$$

and

$$\begin{aligned} C_{\text{bottom}}=\eta_{A,\text{bottom}}\phi_{\text{bottom}}\left( d,\varepsilon,c,\Phi,T \right)A_{\text{bottom}}=\text{UAC}_{\text{bottom}}A_{\text{bottom}}\#\left( S6 \right) \end{aligned}$$

respectively.

Because the two EDLs are connected through the ionically conductive PEE layer, the measured interfacial capacitance can be described as the series combination of $C_{\mathrm{top}}$ and $C_{\mathrm{bottom}}$. A small geometrical electrode–electrode capacitance, $C_{\mathrm{geo}}$, may exist in parallel:

$$\begin{aligned} C_{P}\left( P \right)=\left[ \frac{1}{C_{\text{top}}\left( P \right)}+\frac{1}{C_{\text{bottom}}} \right]^{-1}+C_{\text{geo}}\#\left( S7 \right) \end{aligned}$$

Substituting Equations (S5) and (S6) into Equation (S7) gives

$$\begin{aligned} C_{P}\left( P \right)=\left[ \frac{1}{\text{UAC}_{\text{top}}A_{\text{top}}\left( P \right)}+\frac{1}{\text{UAC}_{\text{bottom}}A_{\text{bottom}}} \right]^{-1}+C_{\text{geo}}\#\left( S8 \right) \end{aligned}$$

or equivalently,

$$\begin{aligned} C_{P}\left( P \right)=\frac{UAC_{top}A_{top}\left( P \right)\cdot UAC_{bottom}A_{bottom}}{UAC_{top}A_{top}\left( P \right)+UAC_{bottom}A_{bottom}}+C_{geo}\#\left( S9 \right) \end{aligned}$$

Equation (S9) directly links the output capacitance of the pressure-sensing channel to the effective contact area at the electrode/PEE interface.

If the two electrode/PEE interfaces have comparable iontronic properties,

$$\begin{aligned} UAC_{top}\approx UAC_{bottom}=UAC\#\left( S10 \right) \end{aligned}$$

Equation (S9) becomes

$$\begin{aligned} C_{P}\left( P \right)=\text{UAC}\frac{A_{\text{top}}\left( P \right)A_{\text{bottom}}}{A_{\text{top}}\left( P \right)+A_{\text{bottom}}}+C_{\text{geo}}\#\left( S11 \right) \end{aligned}$$

Since $A_{\mathrm{bottom}}$ is pressure-independent, the pressure response is governed by $A_{\mathrm{top}}(P)$. When $A_{\mathrm{bottom}}$ is sufficiently large or when $C_{\mathrm{bottom}}\gg C_{\mathrm{top}}(P)$, the series capacitance is dominated by the pressure-sensitive upper interface. In this case, Equation (S9) can be simplified to

$$\begin{aligned} C_{P}\left( P \right)\approx\text{UAC}_{\text{top}}A_{\text{top}}\left( P \right)+C_{\text{geo}}\#\left( S12 \right) \end{aligned}$$

At zero pressure,

$$\begin{aligned} C_{P,0}=\text{UAC}_{\text{top}}A_{\text{top},0}+C_{\text{geo}}\#\left( S13 \right) \end{aligned}$$

where $A_{\mathrm{top},0}$ is the initial effective contact area. Therefore, the pressure-induced capacitance change is

$$\begin{aligned} \Delta C_{P}\left( P \right)=C_{P}\left( P \right)-C_{P,0}=\text{UAC}_{\text{top}}\left[ A_{\text{top}}\left( P \right)-A_{\text{top},0} \right]\#\left( S14 \right) \end{aligned}$$

Equation (S14) is the central contact-area-dependent capacitance relation for the iontronic pressure sensor. It indicates that the measured capacitance change is proportional to the increase in EDL-active contact area, with the proportionality factor determined by the intrinsic UAC of the electrode/PEE interface^3,4^.

Within the calibrated linear working range, the pressure-induced evolution of the upper contact area can be phenomenologically expressed as

$$\begin{aligned} A_{\text{top}}\left( P \right)=A_{\text{top},0}+S_{A}P\#\left( S15 \right) \end{aligned}$$

where $S_{A}=dA_{\mathrm{top}}/dP$ is the contact-area sensitivity of the microstructured PEE layer. Substituting Equation (S15) into Equation (S14) yields

$$\begin{aligned} \Delta C_{P}\left( P \right)=\text{UAC}_{\text{top}}S_{A}P\#\left( S16 \right) \end{aligned}$$

Thus, the output capacitance can be written as

$$\begin{aligned} C_{P}\left( P \right)=C_{P0}+S_{P}P\#\left( S17 \right) \end{aligned}$$

where

$$\begin{aligned} S_{P}=UAC_{top}S_{A}=\eta_{A,top}\phi_{top}\left( d,\varepsilon,c,\Phi,T \right)S_{A}\#\left( S18 \right) \end{aligned}$$

Equation (S18) shows that the pressure sensitivity of the iontronic sensing channel is determined by two coupled factors: the intrinsic unit-area capacitance of the electrode/PEE EDL interface and the mechanical efficiency by which the microstructured PEE layer converts normal pressure into additional contact area.

For the temperature-sensing channel, the output capacitance is also dominated by the iontronic interfacial capacitance arising from the electrical double layer (EDL) formed at the electrode/ionic elastomer interface. As derived in the pressure-sensing section, the EDL capacitance based on the Gouy–Chapman–Stern model can be expressed as

$$\begin{aligned} C_{\text{EDL}}=\left( \frac{1}{C_{H}}+\frac{1}{C_{D}} \right)^{-1}=\eta_{A}\cdot\phi\left( d,\varepsilon,c,\Phi,T \right)\cdot A=\text{UAC}\left( T \right)\cdot A\#\left( S19 \right) \end{aligned}$$

where $C_{H}$ and $C_{D}$ are the Helmholtz-layer capacitance and diffuse-layer capacitance, respectively. $\eta_{A}$ is the interfacial roughness factor, $d$ is the effective Helmholtz-layer thickness, $\varepsilon$ is the dielectric constant of the ionic medium, $c$ is the ion concentration, $\Phi$ is the interfacial potential, and $T$ is the temperature. The term $\phi\left( d,\varepsilon,c,\Phi,T \right)$ describes the combined physicochemical contribution of the iontronic interface, and $\mathrm{UAC}(T)$ represents the temperature-dependent unit-area capacitance.

For the sandwich-structured iontronic temperature sensor, the ionic elastomer is stably sandwiched between the upper and lower electrodes. During temperature sensing, no external mechanical loading is applied, and the electrode/ionic elastomer contact area is assumed to remain unchanged. Therefore,

$$\begin{aligned} A\left( T \right)\approx A_{0}\#\left( S20 \right) \end{aligned}$$

where $A_{0}$ is the constant effective contact area. Under this condition, the capacitance response is mainly governed by the temperature-dependent variation of the interfacial $\text{UAC}$^5,6^, rather than by contact-area variation. Thus, Equation (S19) can be simplified as

$$\begin{aligned} C_{T}\left( T \right)=\text{UAC}\left( T \right)\cdot A_{0}\#\left( S21 \right) \end{aligned}$$

Since the contact area and interfacial geometry are fixed, the temperature-dependent term in Equation (S19) can be expressed as

$$\begin{aligned} \text{UAC}\left( T \right)=\eta_{A}\cdot\phi\left( d,\varepsilon,c,\Phi,T \right)=\text{UAC}_{0}\cdot f\left( T \right)\#\left( S22 \right) \end{aligned}$$

where $\text{UAC}_{0}$ is the unit-area capacitance at the reference state, and f(T) describes the temperature-induced modulation of the EDL. Substituting Equation (S22) into Equation (S21) gives

$$\begin{aligned} C_{T}\left( T \right)=\text{UAC}_{0}\cdot A_{0}\cdot f\left( T \right)\#\left( S23 \right) \end{aligned}$$

Therefore, for the sandwich-structured temperature sensor with a fixed contact area, the output capacitance is directly related to the temperature-dependent EDL property:

$$\begin{aligned} C_{T}\left( T \right)\propto\text{UAC}\left( T \right)\#\left( S24 \right) \end{aligned}$$

Within the calibrated linear working range, $f(T)$ can be approximated by a first-order linear relation around the reference temperature $T_{0}$:

$$\begin{aligned} f\left( T \right)\approx f\left( T_{0} \right)+\left. \frac{df}{dT} \right|_{T_{0}}\left( T-T_{0} \right)\#\left( S25 \right) \end{aligned}$$

Thus, the temperature-dependent output capacitance can be phenomenologically expressed as

$$\begin{aligned} C_{T}\left( T \right)=C_{T,0}+S_{T}\left( T-T_{0} \right)\#\left( S26 \right) \end{aligned}$$

where $C_{T,0}$ is the capacitance at the reference temperature $T_{0}$, and $S_{T}$ is the temperature sensitivity obtained from calibration:

$$\begin{aligned} S_{T}=\text{UAC}_{0}A_{0}\left. \frac{df}{dT} \right|_{T_{0}}\#\left( S27 \right) \end{aligned}$$

This derivation shows that the temperature-sensing channel follows an area-fixed UAC-modulated capacitance mechanism. Different from the pressure-sensing channel, in which the capacitance variation mainly originates from pressure-induced changes in contact area, the temperature-sensing channel relies on the temperature-dependent modulation of the interfacial EDL capacitance while maintaining an approximately constant contact area. Equation (S26) is a local approximation over 25–45 °C (Supplementary Fig. S23), not a full-range linear law. Impedance trends (Supplementary Fig. S24) support temperature-dependent ionic transport without uniquely identifying mobility, dielectric properties or double-layer dimensions.

**Supplementary Note 2. Calibration protocol of the iontronic pressure and temperature sensor array**

**Pressure–Capacitance Calibration Protocol**

The capacitance–pressure calibration of the iontronic tactile sensors was performed using a force-controlled mechanical loading system coupled with an LCR meter. Briefly, the iontronic tactile sensor was fixed on a motorized translation stage, while a digital force gauge/dynamometer (Mark-10, M5-10) was mounted opposite to the sensor to record the applied normal force. A flat silicone rubber pad was attached to the loading head to ensure uniform pressure distribution and to avoid local stress concentration during contact with the sensor surface. The loading head was driven toward the sensor at a constant displacement rate of **1 mm/min**.

During the loading process, the applied force was continuously recorded by the Mark-10 dynamometer, and the corresponding capacitance of the sensor was simultaneously measured using an LCR meter (Tonghui 2840A). The LCR meter was operated in Cs–Rs mode at 10 kHz, with 1 V AC excitation and 100 mV DC bias. The applied pressure ($P$) was calculated according to:

$$\begin{aligned} P=\frac{F}{A}\#\left( S28 \right) \end{aligned}$$

where $F$ is the normal force measured by the Mark-10 dynamometer and $A$ is the effective contact area between the loading pad and the sensor. For the 3 × 3 tactile sensor array, each sensing pixel was calibrated individually by aligning the loading pad with the center of the target pixel^3,4^.

For each sensing unit, the initial capacitance $C_{0}$ was recorded under the no-load condition. The capacitance response under external pressure was expressed as

either the absolute capacitance variation $\Delta C=C-C_{0}$ or the normalized capacitance variation ${\Delta C}/{C_{0}}$. The pressure sensitivity was extracted from the linear fitting of the normalized capacitance–pressure curve:

$$\begin{aligned} S=\frac{{\Delta C}/{C_{0}}}{\Delta P}\#\left( S29 \right) \end{aligned}$$

where $S$ represents the normalized pressure sensitivity. Each calibration test was repeated several times to evaluate the repeatability of the capacitance response. The obtained calibration curves were then used to convert the measured capacitance signals of the tactile sensor array into pressure values.

For the 3 × 3 sensor array, the capacitance–pressure curve of each pixel was fitted independently to obtain its baseline capacitance, sensitivity, and linear fitting coefficient. The pixel-to-pixel variation was compensated by applying an individual calibration factor to each sensing unit. Specifically, the measured capacitance response of each pixel was converted into pressure using its own fitted calibration equation:

$$\begin{aligned} P_{i}=\frac{\left( C_{i}-C_{0,i} \right)/C_{0,i}}{S_{i}}\#\left( S30 \right) \end{aligned}$$

where $C_{i}$, $C_{0,i}$ and $S_{i}$ are the measured capacitance, initial capacitance, and normalized sensitivity of the $i$-th sensing pixel, respectively. The calibrated pressure values of all nine pixels were subsequently reconstructed into a two-dimensional pressure distribution map using MATLAB.

**Temperature–Capacitance Calibration Protocol**

The capacitance–temperature calibration of the iontronic temperature sensor was performed using a programmable temperature-controlled stage coupled with an LCR meter. Briefly, the sensor was fixed on a temperature-controlled stage (MK2000B), and the temperature program was set through the control software. The temperature was varied from 0 to 80 °C with a step interval of 10 °C. The temperature ramping rate was set to the Hold mode, corresponding to the maximum temperature-changing rate of the stage. At each temperature step, the stage was maintained for 10 min to allow the sensor output to reach a stable state.

During the calibration process, the capacitance of the sensor was continuously recorded using an LCR meter. The LCR meter was operated in Cs–Rs mode at 10 kHz, with 1 V AC excitation and 100 mV DC bias. The applied temperature $T$ was defined as the set temperature of the temperature-controlled stage. For each sensing unit, the initial capacitance $C_{0}$ was recorded at the reference temperature $T_{0}$. The capacitance response under temperature variation was expressed as either the absolute capacitance variation $\Delta C=C-C_{0}$ or the normalized capacitance variation $\Delta C/C_{0}$.

A local normalized temperature sensitivity can be defined by a first-order fit over the calibrated local interval:

$$\begin{aligned} S_{T}=\frac{\Delta C/C_{0}}{\Delta T}\#\left( S31 \right) \end{aligned}$$

where $S_{T}$ represents the normalized temperature sensitivity, and $\Delta T=T-T_{0}$ is the temperature variation relative to the reference temperature. Each calibration test was repeated several times to evaluate the repeatability of the capacitance response. The obtained calibration curves were then used to convert the measured capacitance signals of the temperature sensor into temperature values.

For the 3 × 3 sensor array, the local capacitance–temperature curve of each pixel was fitted independently to obtain its baseline capacitance, temperature sensitivity, and linear fitting coefficient. The pixel-to-pixel variation was compensated by applying an individual calibration factor to each sensing unit. Specifically, the measured capacitance response of each pixel was converted into temperature using its own local fitted calibration equation; the relation below is not a global 0–80 °C inverse:

$$\begin{aligned} T_{i}=T_{0}+\frac{\left( C_{i}-C_{0,i} \right)/C_{0,i}}{S_{T,i}}\#\left( S32 \right) \end{aligned}$$

where $C_{i}$, $C_{0,i}$, and $S_{T,i}$ are the measured capacitance, initial capacitance, and normalized temperature sensitivity of the $i$-th sensing pixel, respectively. The calibrated temperature values of all nine pixels were subsequently reconstructed into a two-dimensional temperature distribution map using MATLAB.

After the capacitance–temperature calibration of each sensing unit, the measured capacitance response of the sensor array was converted into temperature or response intensity according to the pixel-specific calibration curve. The two-dimensional temperature distribution was reconstructed using MATLAB. Specifically, the spatial coordinates of the sensing pixels were defined as $x$ and $y$, while the corresponding calibrated temperature values or response intensities were defined as $z$. A dense two-dimensional grid was generated using the meshgrid function, and the discrete sensor responses were interpolated onto this grid using the griddata function with the natural-neighbor interpolation method. The interpolation procedure was used only for visualization of the spatial temperature distribution and did not alter the experimentally measured pixel responses used for quantitative analysis. The reconstructed temperature map was plotted as a continuous surface/heat map using the surf function. Regions outside the interpolation boundary were assigned a value of zero to represent the non-activated background. A fixed color scale was used for different temperature maps to allow direct visual comparison among different thermal patterns.

**Supplementary Note 3. Array mapping and row-wise vectorization**

After the capacitance–pressure calibration of each sensing unit, the measured capacitance response of the tactile sensor array was converted into pressure or response intensity according to the pixel-specific calibration curve.

The two-dimensional pressure distribution was reconstructed using MATLAB. Specifically, the spatial coordinates of the sensing or stimulation points were defined as x and y, while the corresponding calibrated pressure values or response intensities were defined as z. A dense two-dimensional grid was generated using the meshgrid function, and the discrete sensor responses were interpolated onto this grid using the griddata function with the natural-neighbor interpolation method. The interpolation procedure was used only for visualization of the spatial pressure distribution and did not alter the experimentally measured pixel responses used for quantitative analysis.

The reconstructed pressure map was plotted as a continuous surface/heat map using the surf function. Regions outside the interpolation boundary were assigned a value of zero to represent the non-activated background. A fixed color scale was used for different pressure maps to allow direct visual comparison among different tactile patterns. The final pressure maps were exported as high-resolution images for presentation.

For the classification input in Fig. 4, the calibrated 3 × 3 pressure or temperature response matrix was flattened by row-wise vectorization: [x11, x12, x13; x21, x22, x23; x31, x32, x33] → [x11, x12, x13, x21, x22, x23, x31, x32, x33].

**Supplementary Note 4. Classification network and feature definition**

The Fig. 4 dataset comprises nine continuous class recordings with 100 frames per class (900 frames total), rather than independently repeated trials. Pressure-only, temperature-only, and fused inputs were evaluated with the same fixed MLP and the same contiguous-block assignments. Each fold used one 10-frame test block per class; the remaining frames were divided into fitting and inner-validation subsets as specified in Supplementary Table S9.

Pulse and measured PSC records were paired by class and frame index; no common acquisition timestamp was available. Cumulative descriptors were formed before partitioning, normalized using fitting-subset statistics, and supplied to the classifier. The pressure-only and temperature-only inputs contained one modality branch, whereas fusion concatenated both branches.

All conditions used the fixed architecture FC24–ReLU–dropout 0.20–FC12–ReLU–FC9–softmax, Adam optimization with a 0.003 learning rate, batch size 64, 120 epochs, and the documented random seeds. A sensitivity analysis excluded blocks adjacent to the test block while retaining the same network and preprocessing.

Under within-recording contiguous-block validation, PSC pressure-only, temperature-only, and fusion accuracies were 77.56%, 82.00%, and 92.00%, respectively; fused macro-F1 was 0.9215. The adjacent-block exclusion analysis yielded 67.44%, 72.11%, and 82.00%, with fused macro-F1 = 0.8253. These results evaluate held-out frames from the same continuous recordings and do not establish trial-, object-, session-, or day-level generalization.

**Supplementary Note 5. Feed-forward robotic sorting workflow**

In the robotic sorting demonstration, each object was contacted by the flexible sensor patch under a fixed displacement. The pressure channel encoded the reaction force associated with stiffness, while the temperature channel encoded the photothermal temperature state associated with object color. The acquired capacitance/voltage signals were converted into pulse trains, applied to the artificial synaptic device and transformed into PSC features^7^. The predicted class label was then sent to the robotic-arm controller as the sorting command.

The feed-forward demonstration is provided as Supplementary Movie S1. This workflow links fixed-displacement tactile contact, capacitance acquisition, spike encoding, synaptic-current readout, classification and robotic actuation in one signal chain. The classifier operates offline, and no post-actuation measurement is returned for corrective control.

**Supplementary Note 6. Joint pressure–temperature calibration and separate channel-stability measurements**

Joint calibration was performed using a 4 × 4 pressure–temperature matrix (Supplementary Figs. S20 and S21), with three repeated acquisitions per condition rather than measurements from three independent devices. The results show that the pressure response varies with temperature, indicating that temperature compensation is required for quantitative pressure measurement. Leave-one-condition-out validation was subsequently performed by excluding one pressure–temperature condition from model fitting and using the remaining conditions for calibration. Together, these results provide a systematic evaluation of the joint pressure–temperature calibration performance across the full range of conditions included in the measurement matrix. The matrix comprises pressures of 0, 100, 200 and 300 kPa and temperatures of 10, 25, 45 and 60 °C.

The stability of the two sensing channels was evaluated in separate recordings by applying a pressure of 100 kPa at 45 °C for 60 min. The pressure and temperature outputs recorded during this period are presented in Supplementary Figure S22. This combined-loading experiment was conducted separately from the pressure-only stability test; therefore, the previously obtained pressure-channel drift of 0.99% was not used to characterize the results in Supplementary Figure S22. The separate pressure and temperature records yielded drifts of 1.23% and 1.45%, respectively. The 0.99% result was obtained at 80 kPa for 30 min, 25 °C and 50% RH.

**Supplementary Note 7. Grouped-record analysis using pulse and modeled PSC representations**

A separate dataset comprising 27 records was used for offline classification analysis. The dataset contained three records for each of the nine color–stiffness classes. The raw analog signals were first converted into pulse trains, which were subsequently transformed into modeled PSC states using a first-order recurrence model calibrated with separately collected pulse–PSC data. The model parameters were α = 0.938645569620253, gain = 2.50564304117974 × 10⁻⁷, and offset = 9.65979588978323 × 10⁻⁶. Thus, the resulting PSC states represent model-estimated responses rather than PSC signals directly measured from the 27 records.

Seven descriptors were extracted from each channel. Classification was performed using a linear support vector machine within an error-correcting output-codes framework. Three-fold leave-one-replicate-identifier-out validation was used, with 18 records for training and 9 records for testing in each fold. Ridge-parameter selection and z-score normalization were performed using only the training data within each outer fold. With identical folds, fused raw, pulse and modeled PSC-state representations yielded accuracy/macro-F1 of 62.96%/0.600, 40.74%/0.387 and 66.67%/0.667, respectively.

For the Red_20_rep1 record, pressure samples 1300–20000 were paired with temperature samples 1–18701 to form equal-length sequences. Because a common acquisition timestamp was unavailable, this pairing should be regarded as offline signal alignment rather than synchronization during measurement. The replicate identifiers were used to define the validation folds but do not necessarily represent independently fabricated specimens.

**Supplementary Figures**

**Supplementary Figure S1. Module-level signal flow and implementation allocation of the bimodal iontronic afferent system.** The pressure and temperature sensor arrays are read by the capacitance-acquisition circuit and converted into modality-specific pulse trains by the dual-modal spike-encoding circuit. These pulses stimulate the artificial synaptic device, and the resulting postsynaptic-current (PSC) responses are recorded by the current-sampling circuit. The recorded PSC traces are subsequently processed through offline feature extraction, normalization, common multilayer perceptron (MLP) classification after feature concatenation, and decision generation. The predicted class is then transmitted to the robot-hand controller to execute the corresponding robotic action.

**Supplementary Figure S2. Mechanical properties of PEEs prepared with different neutral monomers. (a)** Stress–strain curves of PEE-MA, PEE-BA, and PEE-MEA, showing that PEE-MA exhibits the highest fracture stress, fracture strain, and tensile modulus of 5100 kPa, 167.8%, and 26945.1 kPa, respectively. PEE-BA and PEE-MEA show lower fracture stresses of 547.5 and 230.2 kPa, fracture strains of 102.9% and 53.2%, and tensile moduli of 888.05 and 551.3 kPa, respectively. **(b)** Hysteresis loops of PEE-BA and PEE-MEA, revealing hysteresis ratios of 55.6% and 2.0%, respectively. **(c–f)**, Fracture stress, fracture strain, tensile modulus and hysteresis ratio, respectively. (n = 3).

**Supplementary Figure S3. Capacitance drift of PEEs prepared using different neutral monomers.** **(a–c)**, Representative time-dependent capacitance traces of PEE-MA **(a)**, PEE-BA **(b)** and PEE-MEA **(c)** recorded over 600 s, yielding drift ratios of 2.98%, 1.55% and 0.84%, respectively. **(d)**, Overlaid capacitance traces comparing the temporal stability of the three PEE formulations. **(e)**, Statistical comparison of the corresponding drift ratios. Bars and error bars represent the mean ± s.d., and black dots denote individual measurements (n = 4).

**Supplementary Figure S4. Mechanical properties of PEEs with different ionic monomer-to-neutral monomer ratios. (a)**, Representative tensile stress–strain curves of PEE-1:0, PEE-1:1, PEE-1:2 and PEE-1:3. **(b)**, Representative loading–unloading curves of the four PEE formulations, illustrating their different mechanical hysteresis behaviours. **(c–e)**, Statistical comparison of the fracture stress **(c)**, fracture strain **(d)** and Young’s modulus **(e)** of the four formulations. **(f)**, Corresponding hysteresis ratios calculated from the loading–unloading curves. PEE-1:0 exhibits the highest fracture stress, fracture strain and modulus, whereas PEE-1:2 shows the lowest hysteresis ratio. Bars and error bars represent the mean ± s.d., and black dots denote individual measurements (n = 3).

**Supplementary Figure S5. Capacitance drift of PEEs with different ionic monomer-to-neutral monomer ratios. (a)**, Representative time-dependent capacitance traces of PEE-1:0, PEE-1:1, PEE-1:2 and PEE-1:3 recorded over 600 s. **(b)**, Corresponding drift ratios of the four PEE formulations. The drift ratio decreases from 1.42 ± 0.10% for PEE-1:0 to 0.74 ± 0.07% for PEE-1:3, indicating improved capacitance stability with increasing neutral-monomer content. Data are presented as the mean ± s.d. (n = 3).

**Supplementary Figure S6. Mechanical properties of PEEs with different crosslinking degrees. (a)**, Representative tensile stress–strain curves of PEE-1%, PEE-1.5%, PEE-2%, PEE-2.5% and PEE-3%. **(b)**, Representative loading–unloading stress–strain curves of the five PEE formulations, showing their different mechanical hysteresis behaviours. **(c–f)**, Statistical comparison of the fracture stress **(c)**, fracture strain **(d)**, tensile modulus **(e)** and hysteresis ratio **(f)**. Increasing the crosslinking degree generally increases the tensile modulus while reducing the fracture strain. PEE-3% exhibits the highest fracture stress and tensile modulus together with the lowest hysteresis ratio. Bars and error bars represent the mean ± s.d., and black dots denote individual measurements (n = 3).

**Supplementary Figure S7. Capacitance drift of PEEs with different crosslinker contents. (a)**, Representative time-dependent capacitance traces of PEE-1.5%, PEE-2%, PEE-2.5% and PEE-3% recorded over 600 s. **(b)**, Statistical comparison of the corresponding drift ratios. The mean drift ratios are 1.50 ± 0.18%, 1.38 ± 0.11%, 2.33 ± 0.20% and 0.95 ± 0.05% for PEE-1.5%, PEE-2%, PEE-2.5% and PEE-3%, respectively. PEE-3% exhibits the lowest drift ratio, indicating the highest capacitance stability among the tested formulations. Bars and error bars represent the mean ± s.d., and black dots denote individual measurements (n = 3).

**Supplementary Figure S8.** **Effect of free ion content on the ionic conductivity of PEEs. (a)**, Frequency-dependent impedance spectra of PEEs containing 0%, 5%, 10%, 15% and 20% EMIM-TFSI. The impedance decreases with increasing frequency for all formulations. **(b)**, Statistical comparison of the impedance measured at 1 kHz. The corresponding values are 0.28 ± 0.01, 1.47 ± 0.04, 4.63 ± 0.13, 8.29 ± 0.23 and 30.21 ± 0.71 kΩ for EMIM-TFSI concentrations of 0%, 5%, 10%, 15% and 20%, respectively. Bars and error bars represent the mean ± s.d., and black dots denote individual measurements (n = 3).

**Supplementary Figure S9.** **Cyclic tensile stability of the optimized PEE.** **(a),** Cyclic tensile stress–strain curves recorded at the 1st, 10th, 100th, and 990th cycles show highly overlapped elastic responses within 15% strain, confirming stable mechanical performance without obvious hardening or softening. **(b)**, Mean hysteresis ratios of three independently prepared PEE samples during 1,000 loading–unloading cycles. The hysteresis ratios are 1.13 ± 0.16%, 1.03 ± 0.22% and 1.08 ± 0.32% for samples 1–3, respectively, demonstrating low mechanical energy dissipation and good sample-to-sample reproducibility. Data are presented as the mean ± s.d.

**Supplementary Figure S10. Pressure-dependent impedance characteristics of the PEE material. (a)**, Nyquist plots of the PEE measured under applied pressures of 0, 20 and 50 kPa, showing pressure-dependent changes in the real ((Z')) and imaginary ((-Z'')) components of the impedance. **(b)**, Corresponding Bode plots of impedance magnitude and phase angle as functions of frequency under the same pressure conditions. Solid and dashed curves represent the impedance magnitude and phase angle, respectively. Curves represent the mean, and shaded regions indicate the mean ± s.d. (n = 3).

**Supplementary Figure S11.** **Optical microscopy evaluation of ion leakage in ion gel and PEE.** The materials were examined after applying a pressure of 100 kPa for 30 min. **(a)** The ion gel shows obvious residual traces in the contact region, indicating ion leakage under pressure. **(b)** PEE shows no apparent residue in the contact region, confirming suppressed ion leakage.

**Supplementary Figure S12. EDS characterization of leakage behavior in ion gel and PEE. EDS analysis was performed after applying a pressure of 100 kPa for 30 min. (a)** EDS spectrum of the ion gel, showing pronounced F and S signals on the Cu foil surface. **(b)** EDS spectrum of PEE, showing much weaker F and S signals, suggesting that ion migration is effectively suppressed by immobilization of ionic components within the polymer network^8^.

**Supplementary Figure S13.** **EDS elemental mapping of ion gel and PEE after pressure loading. (a)** EDS elemental maps of the ion gel show obvious F distribution in the contact region, with an F atomic percentage of 8.19%. **(b)** EDS elemental maps of PEE show negligible F distribution in the contact region, with an F atomic percentage of only 0.01%, confirming greatly suppressed ion leakage in PEE.

**Supplementary Figure S14. Comparison of electrical signal drift between PEE and conventional ion gels.** Capacitance drift curves of **(a)** PVA/H₃PO₄ ion gel, **(b)** PVDF ion gel, and **(c)** PEE under 80 kPa for 30 min at 25 °C and 50% RH, showing drift ratios of 1.67%, 6.43%, and 0.99%, respectively.

**Supplementary Figure S15.** **SEM characterization of the microstructured PEE sensing layer.** Overall and enlarged SEM images confirm a well-defined and uniformly arranged microstructure array with clear structural boundaries.

**Supplementary Figure S16. Dynamic response characteristics of the pressure sensor. (a)** Dynamic response and recovery behavior, showing response and recovery times of 39.2 ms and 20.9 ms, respectively. **(b)** Capacitance response under different pressures, showing stable and distinguishable signal outputs. **(c)** Capacitance response under different loading rates, demonstrating reliable dynamic sensing performance with low dependence on loading speed.

**Supplementary Figure S17. Cyclic stability of the temperature sensor over 1000 cycles.** Long-term cyclic temperature response and enlarged insets show stable and repeatable periodic capacitance signals, confirming excellent cycling stability.

**Supplementary Figure S18. Dynamic response of the temperature-sensing channel during a heating–cooling cycle.** Time-resolved capacitance (red; left axis) and measured temperature (blue; right axis) as the temperature was increased from approximately 20.0 to 21.0 °C and subsequently returned to its initial value. The blue and yellow shaded regions denote the heating and cooling intervals, respectively.

**Supplementary Figure S19.** **Response error of the bimodal sensor under coupled temperature-pressure stimuli.** Pressure-induced error of the temperature-sensing channel under increasing pressure shows limited mechanical interference with temperature detection and supports distinguishability within the tested range.

**Supplementary Figure S20.** **Joint pressure–temperature response surfaces. (a)** Capacitance-response surface of pressure sensor. **(b)** Capacitance-response surface of temperature sensor. Both channels were measured across 16 paired conditions comprising pressures of 0, 100, 200 and 300 kPa and temperatures of 10, 25, 45 and 60 °C.

**Supplementary Figure S21. Joint pressure–temperature calibration. (a)** Pressure-channel response for 10, 25, 45 and 60 °C at 0, 100, 200 and 300 kPa; error bars summarize repeated acquisitions. **(b)** Temperature dependence of pressure sensitivity. **(c)** Leave-one-condition-out pressure prediction. **(d)** Leave-one-condition-out temperature prediction. Repeated acquisitions are not independent devices.

**Supplementary Figure S22. Separate one-hour channel-stability records. (a)** Representative pressure-channel capacitance trace. **(b)** Representative temperature-channel capacitance trace.

**Supplementary Figure S23. First-order fit of the temperature-channel response over the local 25–45 °C interval (R² = 0.9705).**

**Supplementary Figure S24. Bode spectra measured at 10, 25, 45 and 60 °C, consistent with temperature-dependent ionic transport.**

**Supplementary Figure S25. Pressure-sensor repeatability.** Repeated pressure calibration curves from three independent tests confirm good sensing repeatability.

**Supplementary Figure S26. Effect of environmental humidity and PDMS encapsulation on the dual-modal sensor responses. (a–b) Normalized capacitance responses (ΔC/C₀) of the temperature-sensing channel measured from 25 to 85 °C at relative humidities (RH) of 50%, 70% and 90% before (a) and after (b) PDMS encapsulation. (c–d) Normalized capacitance responses of the pressure-sensing channel measured from 0 to 200 kPa at 25 °C under the same RH conditions before (c) and after (d) encapsulation.** Compared with the unencapsulated devices, the PDMS-encapsulated devices exhibit substantially reduced response divergence among the three RH conditions, demonstrating effective suppression of humidity-induced interference. Solid lines represent the mean and shaded regions indicate the s.d. (n = 3).

**Supplementary Figure S27.** **Pixel-to-pixel response consistency of the pressure sensor array.** Pressure calibration curves from multiple sensing pixels show reproducible pressure-dependent outputs and confirm good array uniformity.

**Supplementary Figure S28.** **Pixel-to-pixel response consistency of the temperature sensor array.** Temperature-response curves from multiple sensing pixels show consistent temperature-dependent outputs and confirm good array uniformity.

**Supplementary Figure S29. Calibration and pressure-mapping workflow of the 3 × 3 iontronic tactile sensor array.** **(a)** Force-controlled loading and capacitance acquisition setup for the 3 × 3 iontronic pressure sensor array. A Mark-10 dynamometer equipped with a silicone pad was used to apply normal force to each sensing pixel, while the LCR meter recorded the corresponding capacitance output. **(b)** Pixel-specific calibration and linear fitting procedure for the pressure sensor array. Each pixel was calibrated individually to establish the relationship between applied pressure and capacitance response, enabling reconstruction of spatial pressure distributions from the 3 × 3 array. **(c)** Temperature-controlled heating and capacitance acquisition setup for the 3 × 3 iontronic temperature sensor array. The 3 × 3 temperature sensor array was placed on a temperature-controlled stage (MK2000B), and the LCR meter recorded the capacitance response under controlled temperature conditions. **(d)** Pixel-specific calibration and linear fitting procedure for the temperature sensor array. Each pixel was calibrated individually by recording the normalized capacitance response as a function of temperature, enabling reconstruction of spatial temperature distributions from the 3 × 3 array.

**Supplementary Figure S30. Capacitance-to-pulse encoding generated by the gating algorithm.** The upper trace shows the capacitance input used for pulse encoding, with the lower silent threshold and upper maximum-frequency threshold indicated by dashed lines. The lower trace shows the exported gated pulse train generated using 10 ms time bins, a fixed 100 ms high-level duration, silent output below the lower threshold and a linear threshold-window mapping from capacitance to pulse frequency, with the maximum frequency constrained below 10 Hz.

**Supplementary Figure S31. Control classification using encoded-pulse features without synaptic processing. (a) Pressure-only pulse features; (b) temperature-only pulse features; and (c) fused pressure–temperature pulse features.** The corresponding classification accuracies are 35.33%, 49.44%, and 53.44% for the pressure-only, temperature-only and fused pulse representations, respectively.

**Supplementary Figure S32. Nine-class robotic-sorting signal chain. Representative voltage, encoded-pulse and synaptic-current responses are shown for the nine temperature–hardness combinations. T1–T3 denote white, red and black objects, respectively, and H1–H3 denote soft, medium and hard states. These representative signals describe the qualitative feed-forward demonstration and do not constitute repeated physical robotic outcome statistics.**

**Supplementary Figure S33. Temporal integration and relaxation of the synaptic device under pulse-train stimulation. (a)** Baseline-aligned drain-current responses from four measurements, showing progressive current accumulation during stimulation and relaxation after pulse cessation. The dashed curve and shaded region represent the mean and ± standard deviation, respectively. **(b)** Enlarged view of the pulse-induced current accumulation and initial relaxation. **(c)** Pulse-peak current as a function of pulse number, showing cumulative enhancement over ten successive pulses. Data are presented as mean ± standard deviation across four measurements. **(d)** Post-stimulation current relaxation and corresponding exponential fits (dashed lines), with time zero defined as the end of the pulse train. The fitted relaxation time constants are 0.510, 0.530, 0.524, and 0.516 s, with corresponding R² values of 0.981, 0.990, 0.992, and 0.992, respectively.

**Supplementary Figure S34**. Fixed-MLP comparison and validation sensitivity. Under within-recording contiguous-block validation, measured PSC pressure, temperature, and fusion accuracies were 77.56%, 82.00%, and 92.00%, whereas the corresponding pulse-only accuracies were 35.33%, 49.44%, and 53.44%. Adjacent-block exclusion yielded PSC accuracies of 67.44%, 72.11%, and 82.00% and fused pulse accuracy of 36.67%. The fused PSC macro-F1 scores were 0.9215 and 0.8253, respectively.

**Supplementary Tables**

**Supplementary Table S1. Abbreviations, sample names and component definitions used in the manuscript and SI.**

| **Term** | **Definition / role** | **Where used** |
| --- | --- | --- |
| PEE | Polyelectrolyte elastomer used as the iontronic sensing material. | Main text; Supplementary Figs. S1–S20 |
| PEE-MA / PEE-BA / PEE-MEA | PEE formulations prepared with different neutral monomers for material screening. | Supplementary Fig. S2 |
| PEE-1:0 to PEE-1:3 | PEE formulations with different ionic monomer/neutral monomer ratios. | Supplementary Figs. S3–S4 |
| PEE-1.5% to PEE-3% | PEE formulations with different crosslinking degrees. | Supplementary Figs. S6–S7 |
| [VBIM][TFSI] | Ionic monomer component of the PEE network. | Experimental Section; Fig. 2b |
| [EMIM][TFSI] | Ionic liquid/free-ion component used to tune ion transport. | Experimental Section; Fig. 2b |
| MEA | Neutral monomer used in the optimized PEE formulation. | Experimental Section; Supplementary Fig. S2 |
| HDDA | Crosslinker used for PEE network formation. | Experimental Section |
| PI/Cu | Flexible electrode substrate used in sensor and device fabrication. | Experimental Section; Figs. 2 and 3 |
| PDMS | Encapsulation or isolation material for integrated flexible devices. | Experimental Section; Fig. 2a |
| PVDF-HFP/EMIM-TFSI | Ion-gel dielectric layer in the electrolyte-gated artificial synaptic device. | Fig. 3a; Experimental Section |
| β-SnO₂ nanowire | Semiconductor nanowire channel used in the artificial synaptic device. | Fig. 3a–b |
| PPF | Paired-pulse facilitation, a short-term plasticity behavior of the artificial synapse. | Fig. 3d |
| PSC | Postsynaptic current used as the neuromorphic feature output. | Figs. 3 and 4 |
| R² | Coefficient of determination for linear fitting. | Fig. 2d; Methods |

**Supplementary Table S2.** Implementation map of the complete signal-processing and robotic-control workflow.

| Step | Operation | Implementation |
| --- | --- | --- |
| 1 | Pressure and temperature transduction | Iontronic sensor hardware |
| 2 | Capacitance readout | Capacitance-acquisition circuit |
| 3 | Capacitance-to-pulse conversion | STM32 firmware and pulse-output electronics |
| 4 | Synaptic-device stimulation | Physical SnO₂ nanowire synaptic transistor |
| 5 | Pulse-to-PSC temporal transformation | Physical synaptic device |
| 6 | PSC acquisition | Current-sampling circuit |
| 7 | PSC descriptor extraction | MATLAB software |
| 8 | Training-set-based z-score normalization | MATLAB software |
| 9 | Feature concatenation and common MLP classification | MATLAB software |
| 10 | Class decision and command generation | MATLAB software |
| 11 | Sorting-command execution | Robot-control MCU firmware and robotic hardware |

**Supplementary Table S3.** Critical comparison of representative neuromorphic tactile and sensorimotor systems.

| Representative work | Sensory modality and objective | Neuromorphic element | Physical processing and learning | Power characteristic | System/robotic validation | Critical comparison with the present work |
| --- | --- | --- | --- | --- | --- | --- |
| Torricelli and Pace, Small 2026, 22, e11256 | Review of triboelectric neuromorphic touch sensors | Reviews TENG coupling with artificial synapses and neurons | Reviews ex situ, discrete-circuit, direct-gating and monolithic integration; not one experimental classifier | Focuses specifically on self-powered tactile sensing and energy harvesting | Reviews representative tactile systems rather than demonstrating one robotic platform | Establishes self-powered operation as an important direction. The present system instead uses externally powered capacitance acquisition and MCU encoding, but integrates pressure–temperature sensing and cross-modal classification. |
| Belleri et al., Nat. Commun. 2024, 15, 5350 | Artificial-neuron operation in liquid environments; not a tactile-fusion system | Organic mixed ionic–electronic artificial neuron | Physical spike generation, excitability and wetware/biohybrid operation supported by nonlinear simulation and analytical modeling | Externally biased; self-powered operation is not the focus | Bioelectronic and biohybrid neuronal validation | Provides substantially deeper physical modeling of artificial-neuron firing. The present work emphasizes system-level tactile integration rather than biorealistic neuron modeling. |
| Krauhausen et al., Sci. Adv. 2021, 7, eabl5068 | Sensorimotor integration and robot learning | Organic neuromorphic circuit | Physical neuromorphic adaptation supports sensorimotor learning | Self-powered operation is not the primary focus | Learning-enabled robotic maze navigation | Demonstrates stronger adaptive sensorimotor learning. The present system performs feed-forward pressure–temperature recognition and sorting rather than autonomous online learning. |
| Jiang et al., Adv. Sci. 2022, 9, 2106124 | Mechanical tactile recognition, including material hardness and tapping patterns | Proton-gated nanoparticle-assembled synaptic device | Tactile sensing, neural coding and synaptic processing enable device-level recognition without relying on a conventional software classifier | Externally powered portable system | Robotic grasping and tactile-interaction demonstrations | More strongly demonstrates device-level tactile recognition. The present system extends the sensory input to pressure and temperature and performs cross-modal classification, but numerical classification remains offline. |
| Chen et al., Nat. Commun. 2024, 15, 7056 | Artificial tactile afferent nerve and tactile feedback | Organic afferent-nerve devices | Tactile signals are processed within an artificial afferent pathway and incorporated into feedback control | Self-powered operation is not the central claim | Closed-loop tactile feedback for an intelligent robot | Demonstrates a more explicit closed-loop tactile-feedback architecture. The present robotic sorting experiment is a feed-forward perception-to-action demonstration without post-actuation sensory feedback. |
| Krauhausen et al., Nat. Commun. 2024, 15, 4765 | Multimodal sensory learning and behavioral conditioning | Low-voltage organic neuromorphic circuit with synaptic functionality | Local and adaptive integration of multimodal sensory stimuli produces associative learning | Low-voltage operation; self-powered sensing is not claimed | Robot learns to avoid potentially dangerous objects through behavioral conditioning | Demonstrates local adaptive multimodal learning. The present work instead uses physical synaptic-state generation followed by offline cross-modal classification, without autonomous behavioral conditioning. |
| Wei et al., Nat. Commun. 2025, 16, 1060 | Dynamic pressure and stiffness-dependent temporal-tactile processing | Mechano-gated iontronic piezomemristor | Mechanical sensing and excitatory/inhibitory temporal plasticity are integrated in one device; supports Hebbian and BCM-type learning behaviors | Self-powered operation is not claimed | Biohybrid perception–actuation circuit demonstrated as proof of concept | Achieves tighter single-device integration of sensing and memory. The present work uses separate pressure–temperature sensors, an MCU encoder and a synaptic transistor, but supports dual-modal classification and robotic sorting. |
| Chen et al., PNAS 2024, 121, e2407971121 | Chemical/optical signal perception and sensorimotor textiles | Quasi-solid-state iontronic synapse fibers | Multiple fibers provide parallel transmission and spatiotemporal integration of sensory inputs | Femt­ojoule-range synaptic energy consumption; not necessarily a self-powered complete system | Neuromorphic textile controls coordinated motion of artificial fiber muscles | Provides fiber-based parallel processing and substantially lower device-level synaptic energy. The present system targets pressure–temperature tactile fusion and object sorting using a rigid–flexible hybrid electronic platform. |
| He et al., InfoMat 2024, 6, e12493 | Visual–tactile cross-modal perception, memory and pattern recognition | Photoelectric synapse array integrated with a mechanoluminescent layer | Physical bimodal plasticity and associative memory are combined with an artificial neural network | Approximately 20 pJ per artificial perceptual neuron | Visual–tactile pattern-recognition demonstration | Provides compact visual–tactile associative memory. The present work uses pressure and temperature as complementary tactile modalities and adds robotic object sorting, but its classifier is implemented offline. |
| Present work | Pressure–temperature iontronic sensing, tactile classification and object sorting | PEE-based bimodal sensor, MCU spike encoder and electrolyte-gated SnO₂ nanowire synaptic transistor | Physical sensing, pulse encoding and spike-to-PSC transformation; numerical feature extraction, normalization and cross-modal classification are performed offline | Externally powered; the acquisition and encoding subsystem power is reported separately | Feed-forward recognition and robotic sorting of objects with coupled thermal and mechanical properties | The principal contribution is the system-level integration of dual-modal iontronic sensing, modality-specific spike encoding, physical synaptic-state generation, cross-modal classification and robotic action. It does not claim self-powered operation, online learning, closed-loop adaptation or fully hardware-implemented classification. |

**Supplementary Table S4. Main claims and supporting SI evidence.**

| **Main claim / scientific scope** | **Primary main-text location** | **Supporting SI item(s)** | **Purpose** |
| --- | --- | --- | --- |
| PEE formulation is selected through mechanical and electrical screening. | Section 2.2 | Supplementary Figs. S2–S10 | Links formulation choice to mechanics, drift, free-ion content, cyclic stability and impedance. |
| PEE suppresses ion leakage compared with conventional ion gels. | Section 2.2 | Supplementary Figs. S11–S14 | Provides optical, EDS and drift evidence for material stability. |
| Microstructured PEE supports pressure-sensitive iontronic response. | Section 2.2 | Supplementary Fig. S15 | Confirms the morphology and uniformity of the sensing layer. |
| The pressure sensor has fast dynamic response. | Section 2.2 | Supplementary Fig. S16 | Reports response/recovery times and dynamic pressure responses. |
| Temperature sensing remains stable under repeated cycling. | Section 2.2 | Supplementary Fig. S17 | Supports thermal-channel durability. |
| Pressure and temperature channels are distinguishable after calibration. | Section 2.2 | Supplementary Figs. S19–S22 | Addresses thermo-mechanical interference. |
| Pressure response is repeatable across tests. | Section 2.2 | Supplementary Fig. S25 | Supports measurement reproducibility. |
| Sensor arrays show pixel-to-pixel consistency. | Sections 2.2 and 2.4 | Supplementary Figs. S27–S28 | Supports spatial mapping and array-level classification. |
| Dual-feature classification is evaluated using defined metrics. | Section 2.4; Methods | Supplementary Note 4; Supplementary Figs. S31 and S34; Supplementary Tables S9–S10 | Clarifies feature definition, model settings, and class-level performance reporting. |
| Main figures and SI evidence form a complete evidence chain. | All Results sections | Supplementary Table S4 | Provides a concise map of the supporting evidence. |

**Supplementary Table S5.** Consolidated geometric parameters of the bimodal iontronic sensor and electrode array.

| **Component** | **Parameter** | **Value** |
| --- | --- | --- |
| Sensor array | Array configuration | 3 × 3 pixels |
| Sensor array | Nominal pixel diameter | 6.0 mm |
| Sensor array | Nominal projected area per pixel | 28.27 mm² |
| Sensor array | Pixel pitch | 12.0 mm |
| Sensor array | Nominal active-array envelope | 30 × 30 mm² |
| Flexible electrode | Main sensing-section outline | 40 × 50 mm² |
| Temperature-sensing module | Recessed cavity thickness | 100 μm |

**Supplementary Table S6. Consolidated device, sensor and system metrics reported in the manuscript package.**

| **Category** | **Metric / condition** | **Reported value or range** | **Source** |
| --- | --- | --- | --- |
| Pressure sensing | Linearity over broad pressure range | R² = 0.997 from 0 to 300 kPa; R² = 0.999 from 0 to 260 kPa | Fig. 2d |
| Pressure sensing | Signal drift under sustained loading | 0.99% at 80 kPa for 30 min, 25 °C, 50% RH | Fig. 2e; Supplementary Fig. S14 |
| Pressure sensing | Dynamic response/recovery | 39.2 ms response time and 20.9 ms recovery time | Supplementary Fig. S16 |
| Temperature sensing | Stepwise thermal response range in main-text characterization | 0 to 80 °C | Fig. 2g-i |
| Temperature sensing | Long-term cyclic stability | 1,000 heating/cooling cycles | Supplementary Fig. S17 |
| Sensor decoupling | Pressure-induced error in temperature channel | Quantified over the 4 × 4 paired-condition matrix | Supplementary Figs. S19–S21 |
| Sensor array | Pixel-to-pixel consistency | Pressure and temperature array outputs show reproducible pixel responses | Supplementary Figs. S27–S28 |
| Artificial synapse | Repeated transfer sweep stability | 30 consecutive forward/reverse gate-voltage sweeps | Fig. 3c |
| Artificial synapse | Frequency-dependent plasticity | 0.42 to 2.33 Hz input pulse trains | Fig. 3e |
| Artificial synapse | Pulse-duration modulation | 0.21 to 1.9 s | Fig. 3f |
| Artificial synapse | Pulse-amplitude modulation | 0.5 to 3.5 V | Fig. 3g |
| Classification | Dual-feature pressure-temperature recognition | 92.00% accuracy; macro-F1 = 0.9215; within-recording contiguous blocks | Fig. 4; Supplementary Fig. S34; Tables S9–S10 |
| Robotic sorting | Object-state categories | Nine categories combining three colors and three stiffness levels | Fig. 5e-n |

**Supplementary Table S7. Capacitance acquisition and spike-encoding parameters.**

| **Item** | **Rule or value** |
| --- | --- |
| Input channels | Pressure and temperature capacitance channels |
| Offline plotting grid | 10 ms; not the firmware polling interval |
| Pulse high-level duration | 100 ms |
| Lower capacitance thresholds | PCL = TCL = 25 pF |
| Upper capacitance thresholds | PCH = TCH = 500 pF |
| Capacitance-to-frequency mapping | Linear within the threshold window |
| Frequency range | fL = 0.2 Hz; fH = 10 Hz |
| MCU timer rule | ARR = 10000/f − 1; CCR = ARR/5 |
| Polling interval | 100 ms |

**Supplementary Table S8. Power-consumptionbreakdown of the signal-acquisition and spike-encoding subsystem.**

| **Module** | **Power consumption** | **Fraction of total power** |
| --- | --- | --- |
| Capacitance acquisition circuit | ~0.68 mW | ~1.0% |
| Spike-encoding circuit | ~0.46 mW | ~0.7% |
| Power module | ~1.76 mW | ~2.5% |
| STM32 MCU system | ~66.20 mW | ~95.8% |
| Acquisition-and-encoding subtotal | ~69.10 mW | 100% |
| Excluded from subtotal | Synaptic stimulation, PSC sampling, communication, offline classification, command generation, and robotic actuation | Not measured |

**Supplementary Table S9. Classification features, split and model settings.**

| **Setting** | **Documented value** |
| --- | --- |
| Data unit | Nine continuous class recordings × 100 frames = 900 frames; frames are not independent trials |
| Pulse/PSC pairing | Class and frame index; no common timestamp |
| Network | FC24–ReLU–dropout 0.20–FC12–ReLU–FC9–softmax |
| Training | Adam; learning rate 0.003; batch 64; 120 epochs |
| Primary split | Within-recording contiguous blocks; 720 fitting, 90 validation, 90 test frames per fold |
| Sensitivity split | Adjacent blocks excluded from fitting; same test blocks and fixed MLP |
| Normalization | Fitting-subset mean and sample s.d.; zero-variance features removed from fitting data |
| Scope | Within-recording discrimination only; no trial-, object-, session-, or day-level generalization |
| History limitation | Cumulative descriptors formed before partitioning |

**Supplementary Table S10. Overall Fig. 4 classification metrics.**

| **Protocol** | **Representation** | **Pressure accuracy** | **Temperature accuracy** | **Fusion accuracy** | **Fusion macro-F1** |
| --- | --- | --- | --- | --- | --- |
| Within-recording contiguous blocks | Measured PSC | 77.56% | 82.00% | 92.00% | 0.9215 |
| Within-recording contiguous blocks | Encoded pulse | 35.33% | 49.44% | 53.44% | Not used for principal claim |
| Adjacent-block exclusion | Measured PSC | 67.44% | 72.11% | 82.00% | 0.8253 |
| Adjacent-block exclusion | Encoded pulse | Not principal | Not principal | 36.67% | Not principal |

**Supplementary Table S11. Dimensionality-reduction analysis settings.**

| **Item** | **Value** |
| --- | --- |
| t-SNE input representation | Fig. 5 classification feature representation |
| LDA input runs | 60–68 |
| LDA preprocessing | 240-point resampling; 180 augmented traces per class; z-score |
| Random seed | seed 42 |
| LDA regularization | 10⁻³ |
| Projection/evaluation | Two-dimensional LDA; 5-fold 3NN |
| LD information content | LD1–LD8 = 40.59%, 31.44%, 9.59%, 7.38%, 4.22%, 3.03%, 2.27%, 1.48% |

**Supplementary Movie S1. Feed-forward robotic sorting demonstration.** The movie shows the integrated sensing patch contacting objects with coupled color-derived thermal states and stiffness levels, the conversion of pressure and temperature outputs into encoded spike trains and synaptic-current features, and the subsequent robotic sorting action.

**References**

1.	Chang, Y. *et al.* First Decade of Interfacial Iontronic Sensing: From Droplet Sensors to Artificial Skins. *Adv. Mater.* **33**, 2003464 (2021).

2.	Wu, J. Understanding the Electric Double-Layer Structure, Capacitance, and Charging Dynamics. *Chem. Rev.* **122**, 10821–10859 (2022).

3.	Bai, N. *et al.* Graded intrafillable architecture-based iontronic pressure sensor with ultra-broad-range high sensitivity. *Nat. Commun.* **11**, 209 (2020).

4.	Yuan, Y. *et al.* Microstructured Polyelectrolyte Elastomer‐Based Ionotronic Sensors with High Sensitivities and Excellent Stability for Artificial Skins. *Adv. Mater.* **36**, 2310429 (2024).

5.	Lockett, V., Sedev, R., Ralston, J., Horne, M. & Rodopoulos, T. Differential Capacitance of the Electrical Double Layer in Imidazolium-Based Ionic Liquids: Influence of Potential, Cation Size, and Temperature. *J. Phys. Chem. C* **112**, 7486–7495 (2008).

6.	Wang, J., Cui, X., Song, Y., Chen, J. & Zhu, Y. Flexible iontronic sensors with high-precision and high-sensitivity detection for pressure and temperature. *Compos. Commun.* **39**, 101544 (2023).

7.	Yang, J. *et al.* Artificial Synapses Emulated by an Electrolyte‐Gated Tungsten‐Oxide Transistor. *Adv. Mater.* **30**, 1801548 (2018).

8.	Li, C. *et al.* Polyelectrolyte elastomer-based ionotronic sensors with multi-mode sensing capabilities via multi-material 3D printing. *Nat. Commun.* **14**, 4853 (2023).
